# Polyprenylated Acylphloroglucinols from *Hypericum rochelii* and *Hypericum olympicum*—Cytotoxic Effects on Non-Tumorigenic Cell Lines and Antibacterial Potential

**DOI:** 10.3390/ph18101591

**Published:** 2025-10-21

**Authors:** Yana Ilieva, Maya M. Zaharieva, Lyudmila Dimitrova, Mila D. Kaleva, Teodor Marinov, Lili I. Dobreva, Tanya Chan Kim, Zlatina Kokanova-Nedialkova, Iliyan Trayanov, Sofia Titorenkova, Stanislava S. Boyadzhieva, Svetla Danova, Paraskev Nedialkov, Hristo Najdenski

**Affiliations:** 1 The Stephan Angeloff Institute of Microbiology, Bulgarian Academy of Sciences, 1113 Sofia, Bulgaria; illievayana@gmail.com (Y.I.); lus22@abv.bg (L.D.); milakalevavet@abv.bg (M.D.K.); ldobreva@microbio.bas.bg (L.I.D.); tanya_85@abv.bg (T.C.K.); sofiatitorenkova@gmail.com (S.T.); stdanova@yahoo.com (S.D.); hnajdenski@gmail.com (H.N.); 2 Faculty of Pharmacy, Medical University of Sofia, 1000 Sofia, Bulgaria; t.marinov@pharmfac.mu-sofia.bg (T.M.); zlatina.kokanova@pharmfac.mu-sofia.bg (Z.K.-N.); pnedialkov@pharmfac.mu-sofia.bg (P.N.); 3 Institute of Chemical Engineering, Bulgarian Academy of Sciences, 1113 Sofia, Bulgaria; iliyantrayanov@gmail.com (I.T.); maleic@abv.bg (S.S.B.)

**Keywords:** *Hypericum*, antibacterial, non-tumorigenic cell lines, MRSA biofilm genes, *Streptococcus mutans*, lactobacilli, apoptosis, autophagy, CYP450, rabbit skin test

## Abstract

**Objectives**: Research on the antimicrobial effect of *Hypericum* plant constituents is very rarely accompanied by studies of the cytotoxic effect on cell lines. In the current study, besides microbiological tests, an investigation of the cytotoxicity of *Hypericum* active ingredients on five non-tumorigenic cell lines, as well as research into the effect on other factors of host homeostasis, was performed. **Methods**: The main methods applied included an MTT assay, the broth microdilution method (BMD), real-time PCR, live cell imaging with Hoechst dye, Western blot, an enzyme-linked immunosorbent assay (ELISA), and skin irritation test on rabbits. **Results**: The mean inhibitory concentrations (IC_50_) of six selected agents—previously phytochemically characterized extracts and compounds—ranged from 0.63 to 48 µg/mL. Due to their strong antimicrobial effect and favorable cytotoxic profile, the extract RochC from *Hypericum rochelii* and the compound olympiforin B from *Hypericum olympicum* were selected for subsequent studies at their previously determined minimum inhibitory concentrations (MICs) against *Staphylococcus aureus*—0.625 and 1 µg/mL, respectively. These doses were lower than their IC_50_ values and the maximum tolerated concentrations (MTCs), according to ISO 10993-5, Annex C, for fibroblast cells, including a human gingival line. The MIC values of RochC and Olympiforin B against the cariogenic *Streptococcus mutans* were 6 and 3 µg/mL, respectively, values lower than the IC_50_ values of the gingival cells. Olympiforin B inhibited the gene expression of the staphylococcal biofilm-related genes *ica*A and *ica*D, while RochC induced *ica*A and had a versatile effect on *ica*D. The MIC values for lactobacilli strains were higher than for *S. aureus*. The phytoconstituents did not cause cytopathic effects or apoptosis in CCL-1 fibroblasts at 2 × MIC. However, the agents at 1 × MIC significantly induced Atg5 and Atg7, proteins related to autophagy. Cytochrome P450 was not induced in liver cells, with the exception of a dose of 2 × MIC of RochC. The agents did not irritate rabbit skin in vivo at a dose of even 10 × MIC. **Conclusions**: The extract and compound have potential for further pharmacological development.

## 1. Introduction

Antimicrobial resistance (AMR) is reported as a top priority health threat by the World Health Organization (WHO), the G7, and G20 [1] and there is an urgent need for the development of novel antimicrobial agents [2]. Nevertheless, the best efforts of synthetic library chemistry have not led to an increase in the rate of new chemical entities reaching the market [3]. Thus, natural products remain an important source of complex structures for antimicrobial drug leads [4,5].

Interestingly, reports state that while in the West medicinal plant use is steadily rising [6], in Eastern Europe the rate of increase is higher. It has been stated that in Eastern Europe more than 60% of prescriptions contain plant-based drugs; thus, traditional medicine seems to have amalgamated with modern medicine [7]. That tradition drives and necessitates scientific research on native flora for potential medicinal plants, and lesser known species from the genus *Hypericum* L. are potential candidates.

Plants from the *Hypericum* L. genus (Hypericaceae) have traditionally been used for wounds, burns, and ulcers, largely because of their antimicrobial effects [8]. The most popular one, *H. perforatum* L. (St. John’s Wort), has one more distinct property. It has been approved in some European countries for the treatment of depression [9]. Notably, its antidepressant effect seems partly to be connected to restoration of gut microbiota [10].

The antimicrobial effect of *Hypericum* representatives, e.g., *H. perforatum* [11], *H. androsaemum* L., [12], *H. tetrapterum* Fr., [13], *H. roeperianum* Schimp. ex A. Rich. [14], etc., has been well-documented, including in reviews [15,16]. An extract from *H. perforatum* has been patented as a food preservative in the United States [17]. A preparation from St. John’s Wort by the name novoimanine has even been used as a clinical drug in Russia to treat infections [4,18]. The genus and its phytoconstituents have other bioactivities, such as antitumor [19,20,21,22,23,24], antioxidant [12,15,19,20,25], anti-inflamatory [19,26], etc. Hyperforin (HF) is the main antidepressant and antibacterial compound in *H. perforatum* [4]. It is a polycyclic polyprenylated acylphloroglucinol (PPAP) and numerous PPAPs with antimicrobial, cytotoxic, and other activities have been isolated so far from *Hypericum* plants [27,28,29,30,31]. Examples of reviews describing their effect can be found in [16,32,33].

Recently, a number of constituents from perennial *Hypericum* species growing in Bulgaria were isolated, and the antibacterial activity of several of them turned out to be remarkable. This applies to PPAPs from *Hypericum olympicum* L., for extracts and fractions from *Hypericum hirsutum* L., and for extracts from *Hypericum rochelii* Griseb. ex Schenk. The most active principles were phytochemically characterized through ultra-high-performance liquid chromatography–high-resolution mass spectrometry (UHPLC–HRMS) and found to contain polyprenylated phloroglucinols [4]. Most notably, the RochC extract, obtained by supercritical CO_2_ extraction from *H. rochelii*, showed antibacterial activity against *Staphylococcus aureus* as great as the best extract from a *Hypericum* plant so far—an extract from *H. perforatum* with a minimum inhibitory concentration (MIC) of 0.625 µg/mL against a caries-causing *Lactobacillus* sp. [34]. The agents also turned out to have anti-biofilm effects against methicillin-resistant *S. aureus* (MRSA).

These findings are promising, as in the last two decades *S. aureus* and MRSA have been among the main human pathogens and their infections have become more dangerous and expensive to treat due to their increasing AMR [35,36]. Pathogenic Gram-positive bacteria such as *S. aureus*, *Corynebacterium*, etc., cause a diverse range of infections, from mild tonsillitis and pharyngitis to endocarditis, meningitis, septicemia, and toxic shock syndrome [37].

Biofilms are notorious for causing chronic infections in the sites of colonization (wounds, urinary tracts, etc.) [38]. As discussed before, bacterial biofilms contribute to >80% of all infections and 65% of infections in developed countries [35,39]. MRSA, together with *Pseudomonas aeruginosa*, is the most ubiquitous pathogen in nosocomial biofilms [40]. Staphylococci, particularly *S. aureus* and *Staphylococcus epidermidis*, are the most frequent causes of device-associated infections and biofilms [41]. MRSA biofilm colonizes catheters, prosthetic joints, pacemakers, and other devices [38]. This process may lead to implant failure, localized tissue destruction, chronic infection, e.g., bacteremia, or other tissue colonization [41]. MRSA is more frequently associated with mortality than other bacterial infections, especially in patients with wounds and necrotic tissue [35,36,39,42].

The mechanical biofilm barrier between drugs, host immune factors, and the inner biofilm bacterial cells, as well as the dormant cells within, are other mechanisms of AMR, which increases several hundred times in the biofilms of many bacterial species [43]. In addition, the direct bactericidal action of antimicrobials causes more AMR than the bacteriostatic action, or the property of targeting some virulence factor such as biofilm formation [44]. All this makes *S. aureus* and MRSA biofilms promising targets for antimicrobial therapy.

While numerous studies are devoted to the in vitro cytotoxic and anticancer activity of active ingredients from *Hypericum* [19,20,21,22,23,24], they are not always accompanied by examination of the direct toxic effect on normal cell lines. Research into their other effects, such as antimicrobial, antioxidant, antiviral, etc. [11,12,15,19,20,25], almost always lacks such studies. Examples of works focused on the effect on normal cell lines are [20,24,45,46,47,48]. In addition, despite its promising antimicrobial properties, the data on the safety profile of *Hypericum* with respect to beneficial microbiota are also limited and rather underexplored. This is particularly relevant for lactobacilli, which are commonly used probiotic bacteria and key components of the human microbiota [49]. Established antibiotics, e.g., penicillin and tetracycline, often collaterally damage the favorable human microbiota, including the lactobacilli [50,51]. Thus, an important consideration for natural product formulations is to be as harmless as possible to both the patient and their normal microbiota functions.

A complex evaluation of different aspects of the biological activity of *Hypericum* constituents was performed. It aimed to complement their proven effect against dysbiotic pathogens in direct relation to other factors responsible for the body’s healthy homeostasis. Additional antimicrobial research, including the cariogenic *S. mutans* and gene expression related to staphylococcal biofilm formation, turned out to be promising. A cytotoxic study of selected *Hypericum* agents on non-tumorigenic cell lines led to a consistent and building assessment where just two agents, an extract with known phytochemical content and a compound, with an optimal antibacterial and cytotoxic profile, were selected to be tested further. They were studied for cytopathic effect and the induction of apoptosis and autophagy in normal cells at relevant therapeutic antistaphylococcal concentrations, but only autophagy was detected. Other factors in the host system were examined. The agents did not harm beneficial Lactobacillales representatives of human and dairy origin, nor did they induce the cytochrome P450 system (with one exception) at those concentrations. The extract and the compound did not irritate rabbit skin in vivo, even at concentrations ten times higher. The two phytoconstituents have potential for further pharmacological development to treat infections caused by bacterial strains sensitive to them, but more research is warranted to show their full therapeutic potential.

## 2. Results

### 2.1. Experiments with a Broad Panel of Active Ingredients from Hypericum Species

#### 2.1.1. Antibacterial Activity Against Pathogenic Bacteria—Supplementing the Existing Data

In order to broaden and enrich the published research involving several extracts, fractions, and pure compounds from a number of *Hypericum* species growing in Bulgaria [4,29], several experiments were performed. While the MIC values of the PPAPs hyperpolyphyllirin/hyperibine J, olympiforin A, and olympiforin B against several bacteria had been published [29], their minimal bactericidal concentrations (MBCs) were unknown. These values are presented in Table 1.

The *Hypericum* constituents are often not very effective against Gram-negative bacteria, possibly due to their outer membrane [14,52,53]. For that reason, only two agents that were in large enough amounts were tested against *P. aeruginosa* PAO1. RochD is a dichloromethane extract from *H. rochelii*, containing polyprenylated phloroglucinols. The results are presented in Table 2 and are another confirmation that polyprenylated phloroglucinols from *Hypericum* often do not have a significant effect against Gram-negative bacteria.

Filling some gaps in the existing knowledge, the MIC values against *Staphylococcus aureus* of the agents RochD and HirDM100 were refined to be 4.9 and 9.8 µg/mL (mg/L). The MIC of RochD against *Enterococcus faecalis* was refined to 4.9 µg/mL.

#### 2.1.2. The MBC/MIC Ratio Points to Bacteriostatic Activity for Most of the Agents

According to Hlima et al. [54], an agent is considered bactericidal if the MBC-to-MIC ratio is below or equal to four (MBC/MIC ≤ 4) and bacteriostatic if MBC/MIC > 4. Following these instructions, the ratio was calculated for the agents from [4,29], where possible, including some results for the caries-causing bacteria *Streptococcus mutans* [55] (Table 3).

Except for RochCM for *S. aureus* and olympiforin B for *S. mutans*, all other agents were bacteriostatic for the tested bacteria. Being bacteriostatic can be a beneficial quality, as bactericidal antibiotics and antimicrobials are more prone to elicit resistance [44].

#### 2.1.3. Total Activity of the Tested Extracts and Fractions According to Eloff [mL/g]

Table 4 shows the calculated total activity of the extracts and fractions according to the method proposed by Eloff (2000) [6], which takes into account how much can be extracted from a given plant or natural source. The amount extracted from the plant [expressed as mg/g of dried plant material] was divided by the MIC value [mg/mL]. The unit [mL/g] indicates the degree to which one gram of plant material can be diluted and still inhibit the growth of the tested microorganism.

The table shows that RochC, which has previously been found to have an exceptionally high antibacterial effect, again leads in total activity, followed by HirDM90 and RochD. In some cases, the MIC values were similar, but the quantity of material extracted from the plants differed. Thus, *H. rochelii* and *H. hirsutum* are the most promising plants for ethnobotanical use, and finding such plants is one of the main goals of calculating total activity.

#### 2.1.4. Cytotoxicity on Normal Cell Lines—Mean Inhibitory (IC_50_) and Maximum Tolerated Concentrations (MTCs)

The half-maximum inhibitory concentrations, also known as the mean or median inhibitory concentrations (IC_50_), of six agents selected for their strong antibacterial activity were determined. A 3-(4, 5-dimethylthiazolyl-2)-2, 5-diphenyltetrazolium bromide (MTT) assay was used. The results are presented in Table 5 and Table 6.

The results showed that the human embryonic kidney-293 (HEK-293) cell line was the most sensitive, followed by HaCaT, CCL-1, and HEPG2. The human gingival fibroblast (HGF) cell line was the most resilient one.

Table 7 shows the maximum tolerated concentrations (MTCs) of the agents. According to ISO 10993-5 [56], this is the concentration at which at least 70% of the tested cells are viable. An agent has therapeutic potential if its MIC value for a certain bacterial species is lower than its MTC value for a certain cell line.

For RochC, that was the case for *S. aureus* and all the cell lines except HaCaT, and for *E. faecalis* for HGF and HEPG2. RochD was promising for HGF and HEPG2 against *S. aureus* and *E. faecalis*. For HirDM90, that was valid only for *S. aureus* for HGF and HEPG2. Hyperpolyphyllirin/hyperibine J was promising for HGF against *S. aureus*, MRSA, and *E. faecalis*, and for CCL-1 and HEPG2 against *S. aureus*. Olympiforin A showed promise for HGF against all bacteria, and for CCL-1 and HEPG2 against *S. aureus*. Olympiforin B was promising for HGF against all bacteria and for CCL-1 and HEPG2 against *S. aureus* and MRSA.

#### 2.1.5. Results from the Colony-Forming Unit Assay

Three of the least cytotoxic in the MTT assay and most promising agents were selected for each cell line with concentrations of 1/2 × IC_50_, IC_50_, and 2 × IC_50_. The results are presented in Table 8.

Most concentrations of 1/2 × IC_50_ stimulated clonogenicity (except for RochD for HaCaT and olympiforin B and hyperpolyphyllirin/hyperibine J for HEK-293). In three out of ten cases, there was stimulation even at the IC_50_ concentrations (hyperibine J and RochC for HaCaT and again RochC for CCL-1). For two agents, stimulation was also observed even at the 2 × IC_50_ dose (hyperpolyphyllirin/hyperibine J for HaCaT and RochC for CCL-1). In some cases, the 2 × IC_50_ concentrations, despite producing fewer colonies than in the untreated control, had almost no difference in colony number compared to the IC_50_ dose or even slightly exceeded it (RochD for HaCaT and HEK-293 and olympiforin B and hyperpolyphyllirin/hyperibine J). Therefore, the results show that hyperpolyphyllirin/hyperibine J and RochC do not impair the clonogenic ability of the HaCaT and CCL-1 lines, respectively, but stimulate it, even at doses of 2 × IC_50_. RochC does not harm the clonogenicity of HaCaT cells at the IC_50_ dose, only at 2 × IC_50_. Most of the tested agents, selected as the least cytotoxic, stimulate the clonogenicity of HaCaT, HEK-293, and CCL-1 lines at doses of 1/2 × IC_50_.

Two of the cell lines in this study, HEPG2 and HGF, were not suitable for the CFU test, and this is elaborated on in the Discussion.

### 2.2. Selection of Agents with Optimal Activity Profiles and of Cell Lines for Further Experiments

Based on the antibacterial activity, the IC_50_ and the MTC values, in addition to the CFU assay, we selected the extract RochC and the compound olympiforin B for further studies. They are the agents with an optimal activity profile and ratio of antibacterial activity to cytotoxicity. They have outstanding antibacterial activity [4] according to Eloff [57] and relatively low and satisfactory toxicity to eukaryotic cells. In the further experiments, RochC and the compound olympiforin B were most often applied at their MIC concentrations for *S. aureus*—0.625 and 1 µg/mL (mg/L), respectively.

The IC_50_ values of the two agents were higher for all five cell lines than these MIC values (except for olympiforin B in HEK-293). Therefore, at therapeutically relevant concentrations for staphylococcal infections, they would likely be safe for eukaryotic cells in the human or in a veterinary patient. The MTC values were higher for these agents, mostly for the CCL-1 and HGF cell lines. That proved useful, because CCL-1 is the recommended line in ISO 10993-5 [56]. Therefore, this cell line was used for live-cell imaging and Western blot (HGF was also used for the Western blot in addition).

RochC is an extract obtained by supercritical CO_2_ extraction from *H. rochelii*, as mentioned, and has been found to contain some prenylated phloroglucinols, which generally have high antibacterial activity [58], such as olympiforin A [29], hyperpolyphyllirin/hyperibine J, and maculatoquiones A–D [4]. The species that they are found in are listed in the Discussion.

**Figure 1:**
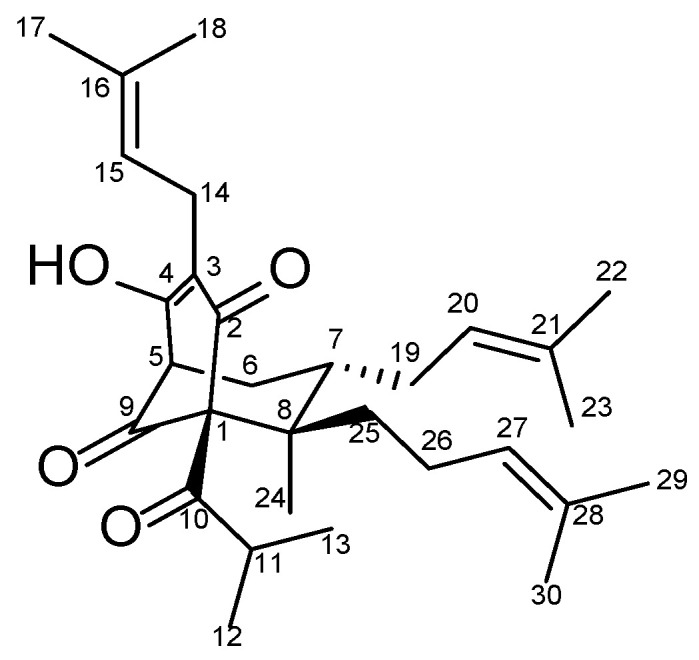
Structure of olympiforin B.

Olympiforin B (Figure 1) is an antibacterial PPAP found in *H. olympicum* L. [29].

### 2.3. Experiments with the Selected Agents RochC and Olympiforin B

#### 2.3.1. Results from the Time Kill Assay

**Figure 2:**
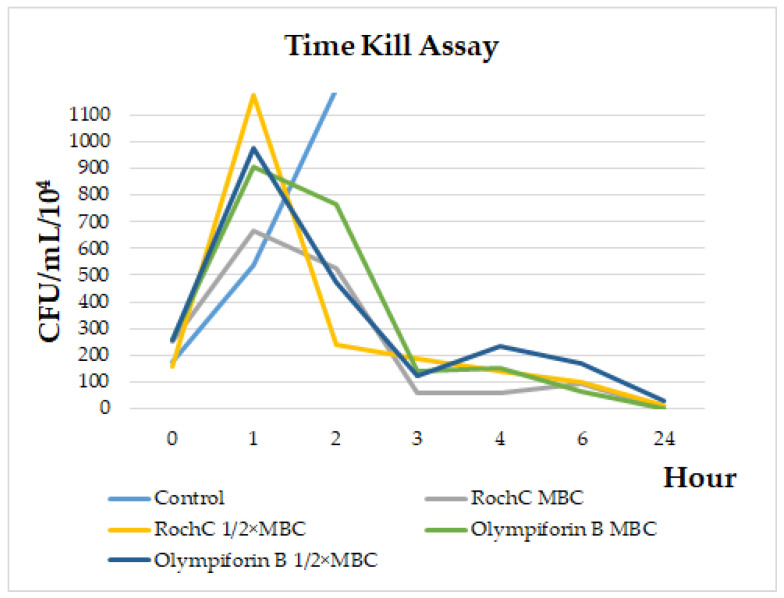
Time kill assay for RochC and olympiforin B on *S. aureus*.

In order to study the dynamics of the bacteriostatic and bactericidal action of the agents, a time kill assay with *S. aureus* was performed. Figure 2 shows that after a peak in bacterial growth in the first hour after treatment, a decline in their number was observed, most sharply between the first and third hours. By 24 h, the MBC concentrations killed all bacteria, while 1/2 × MBC doses reduced the bacteria to 124,000 CFU/mL (RochC) and 302,000 CFU/mL (olympiforin B). The MBC concentrations did not always have fewer bacteria than the 1/2 × MBC doses.

#### 2.3.2. RochC and Olympiforin B Had Very Low MIC and MBC Values Against the Cariogenic *S. mutans*

Because of the promising results and relatively gentle effect of the two agents on gingival fibroblasts (the least cytotoxic in this study), a test against the cariogenic *S. mutans* was performed. Table 9 shows that the two agents inhibited one of the main culprits for dental cavities with very low concentrations for MICs and MBCs. The MIC value of RochC was below 20 μg/mL, once again nominating this extract with an outstanding activity against a pathogen, according to Eloff’s criteria [57]. Olympiforin B, in this case, turned out to be one of the few agents in this study with a bactericidal mode of action, but not bacteriostatic. It can also be noted that their MIC values and even the MBC value of olympiforin B are lower than their IC_50_ and MTC values for the human gingival fibroblasts HGF. The MIC of the compound was five times lower than the MTC and almost six times lower than the IC_50_ for the gingival cells. Therefore, the agents are promising for inhibitory treatment of that cariogenic bacterium and have the potential to be developed as oral care products.

#### 2.3.3. The Antibiofilm Test Against Staphylococci Showed Stronger Activity Against MRSA

The antibiofilm activity of RochC against MRSA and *S. aureus* and the antibiofilm activity of olympiforin B against *S. aureus* (it has already been tested against MRSA [29]) were assessed. Table 10 shows that the MRSA biofilm was more sensitive to the agents, with minimum biofilm inhibitory concentration (MBIC)—resulting in no visible biofilm—lower than their MIC values. The MBIC of RochC was even one-eighth of the MIC value. The median biofilm inhibitory concentrations (MBIC_50_)—or the dose resulting in a reduction in the biofilm by half—were much below the corresponding MIC values for both agents and both bacteria.

**Figure 3:**
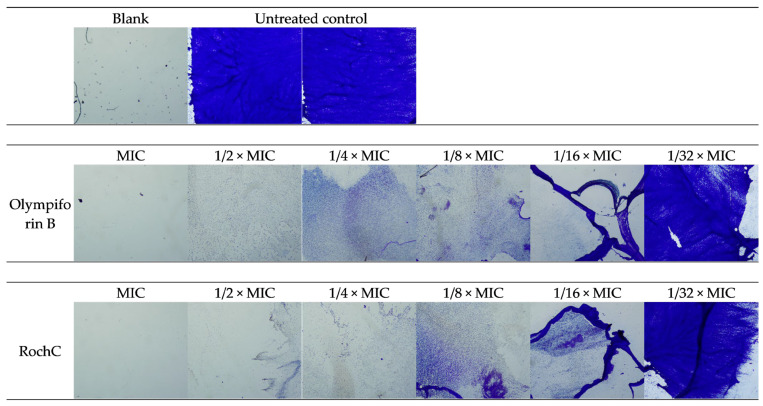
Microscopic images of *S. aureus* biofilm inhibition—40× magnitude.

**Figure 4:**
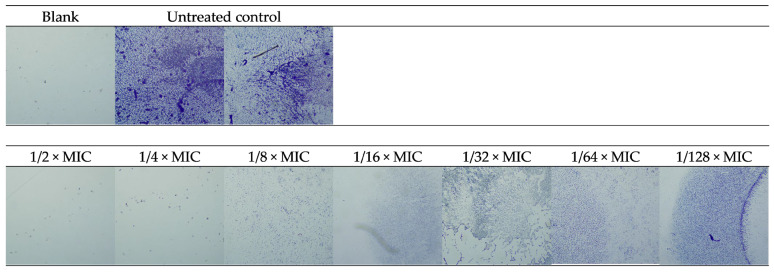
Microscopic images of biofilm inhibition of RochC on MRSA—40× magnitude.

**Figure 5:**
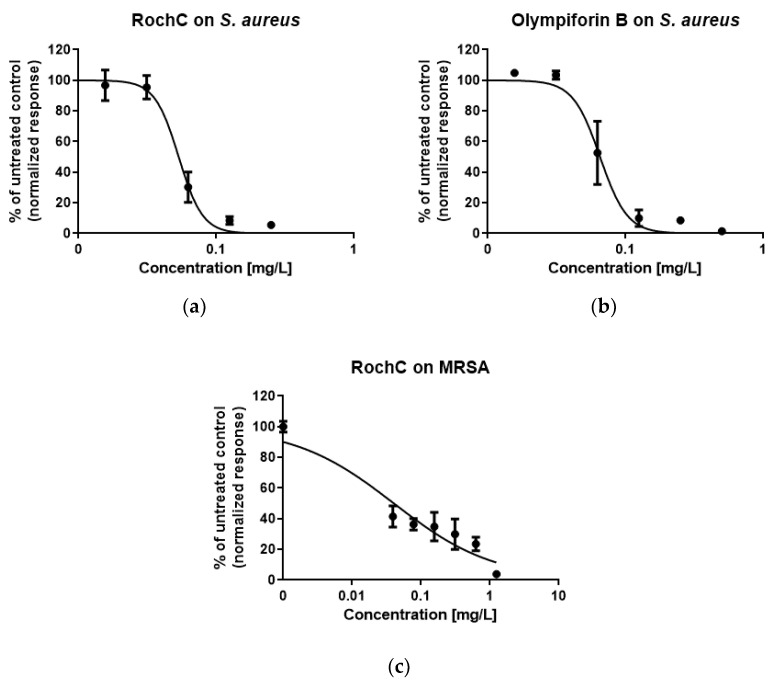
Sigmoidal graphs for biofilm inhibition of (**a**) *S. aureus* after exposure to RochC; (**b**) *S. aureus* after exposure to olympiforin B; (**c**) MRSA after exposure to RochC.

The photo-documented effect on the biofilm is presented in Figure 3 and Figure 4. The sigmoidal graphs associated with the MBIC_50_ calculation are shown in Figure 5.

#### 2.3.4. Olympiforin B Inhibited the Expression of Biofilm-Related Adhesion Genes in MRSA

The more sensitive bacterial strain, MRSA, was chosen for subsequent assessment of the expression of genes related to biofilm formation—*ica*A and *ica*D. The real-time PCR (Table 11) showed that olympiforin B inhibited the relative gene expression of both genes. RochC induced *ica*A, while *ica*D was inhibited by a dose of 1/16 MIC and induced by the lower concentration of 1/32 MIC.

The visible MRSA biofilm was suppressed by a lower dose of olympiforin B (MBIC 0.5 µg/mL) compared to RochC (MBIC 1.25 µg/mL). However, for the compound, that corresponds to 1/2 × MIC in comparison with 1/8 × MIC for the extract RochC. Nevertheless, the biofilm-inducing genes *ica*A and *ica*D were more suppressed by the compound, despite its MBIC not being that much lower than its MIC value, in comparison to RochC.

#### 2.3.5. Effect on Candidate Probiotic Lactobacilli

The bacterial viability of six pre-selected candidate probiotic gut and skin-associated Lactobacilalles strains was estimated in the presence of the two agents.

**Figure 6:**
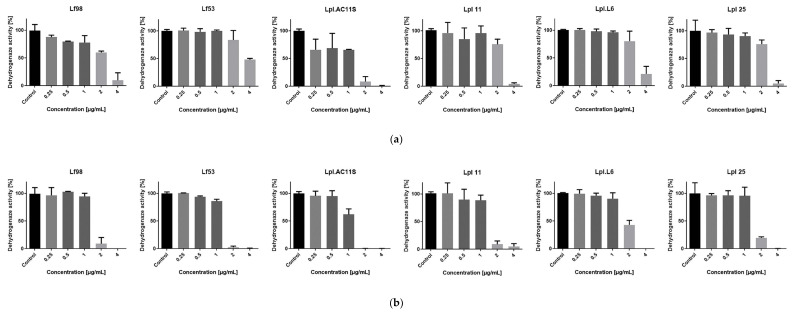
Metabolic (dehydrogenase) activity of lactic acid bacteria treated with the chosen agents: (**a**) RochC; (**b**) olympiforin B.

Table 12 shows that the two agents had MIC values for *Limosilactobacillus fermentum* and *Lactiplantibacillus plantarum* higher than the MIC values for *S. aureus*, implying that they are not a threat to the normal and beneficial microbiota if applied at the anti-staphylococcal therapeutic concentration. The MICs were 2 and 4 µg/mL and even higher than 4 µg/mL for Lf53. Therefore, the MIC of RochC for that strain was a concentration greater than six times its MIC for *S. aureus*. For the other strains, the MICs were two, three, four, and six times greater than the MIC for *S. aureus* of the agents. Although these values are not particularly high, they show that the agents are gentle to lactic acid bacteria in a narrow interval of therapeutic concentrations. Figure 6 shows the dehydrogenase activity of the tested strains treated with the two agents, which can also be considered metabolic activity.

#### 2.3.6. No Cytopathic or Apoptogenic Effect in CCL-1 Was Caused by the MIC and 2 × MIC Doses of the Agents, as Observed with the Help of Live-Cell Imaging

The cells were treated with the low concentrations, therapeutically relevant for *S. aureus*, of RochC and olympiforin B (MIC and 2 × MIC), as well as with a higher concentration of HirDM90 for comparison (IC_50_ and 2 × IC_50_ for the respective cell line), as well as with daunorubicin and hypertonic buffer as positive controls.

**Figure 7:**
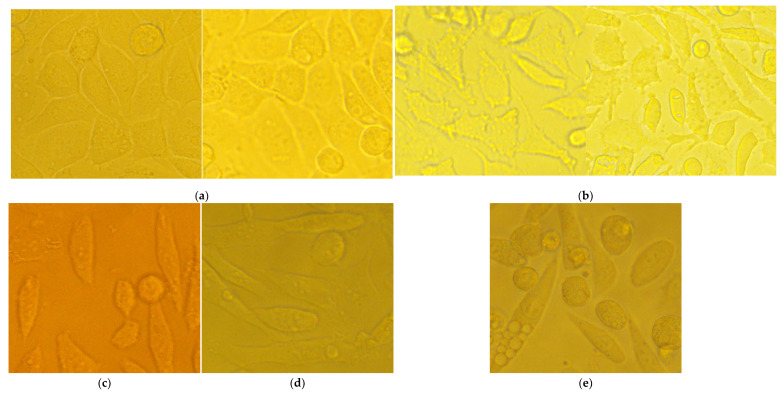
Cytopathic effect of chosen samples with magnification of 400×: (**a**) untreated control; (**b**) only cells treated with hypertonic buffer show increased number of apoptotic blebs on the cell surface; (**c**) HirDM90, dose of 2 × IC_50_, 24 h’ exposure; (**d**) HirDM90, dose of 2 × IC_50_, 48 h exposure; (**e**) HirDM90, dose of 2 × IC_50_, 72 h exposure.

Notably, there was no cytopathic effect with RochC and olympiforin B for all time points applied at concentrations of MIC and 2 × MIC for *S. aureus*. There was a cytopathic effect only associated with the *Hypericum* agent applied at concentrations of IC_50_ and 2 × IC_50_ (HirDM90) and with the hypertonic solution (Figure 7).

Remarkably, apoptotic blebs (membrane bulges typical for apoptotic cells), seen with the higher 400× magnification, were associated only with the hypertonic solution and not with the high dose of HirDM90 at any time point of its exposure.

Summarized, the cytopathic effect was expressed as:

- Apoptotic blebs—in CCL-1 at the hypertonic solution application only.
- Antiproliferative effect (fewer cells in comparison with the untreated control)—at the HirDM90 treatment only; however, the protocol for hypertonic buffer did not allow long enough exposure to check for this effect.
- A rise in the fraction of round, unattached cells—seemed more pronounced with the *Hypericum* agent.
- Shrinkage of the cells and their separation from each other, so that they no longer bordered each other, and the cells, probably as a result, acquired a more irregular and polygonal shape compared to their previous more spindle-like shape—at the hypertonic buffer treatment.

**Figure 8:**
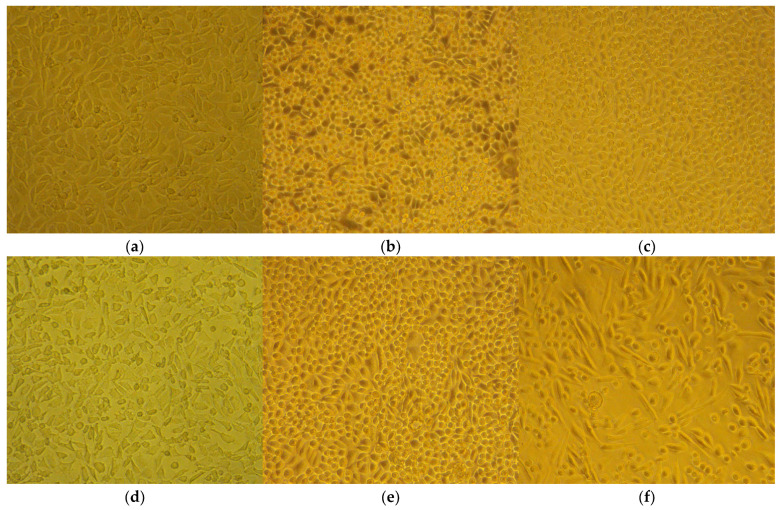
Cytopathic effect after exposure to hypertonic buffer (2 h) or to *Hypericum* agents (72 h) with 100× or 200× magnification. The MIC values are for *S. aureus*. The different concentrations of seeding required two separate controls for the buffer and for the *Hypericum* agents. The panels are the following: (**a**) untreated control for the hypertonic buffer test; (**b**) untreated control for *Hypericum* testing; (**c**) RochC, dose of 2 × MIC; (**d**) hypertonic buffer; (**e**) olympiforin B, dose of 2 × MIC; (**f**) HirDM90, dose of 2 × IC_50_.

The cytopathic effect of HirDM90 was concentration- and time-dependent. At 24 h after seeding, there was already a cell monolayer, and a cytopathic antiproliferative effect was observed only for HirDM90 at the 2 × IC_50_ dose, in the form of fewer cells in some fields of view. At 48 h and even more so at 72 h, the untreated control was characterized by a second layer of rounded cells growing on the lower cell monolayer (in many places at 48 h and everywhere at 72 h). At hour 48, only the treatment with HirDM90 2 × IC_50_ resulted in a clearly distinguishable effect—very sparse, rounded cells, growing on the single-cell monolayer. At hour 72, there was already a clear cytopathic effect with HirDM90 IC_50_ with the same manifestation (Figure 8). Therefore, a cytopathic effect was observed only at doses of IC_50_ and 2 × IC_50_ of HirDM90, with the former at hour 72 and conditionally at hour 48, and with the latter from hour 24 onwards.

**Figure 9:**
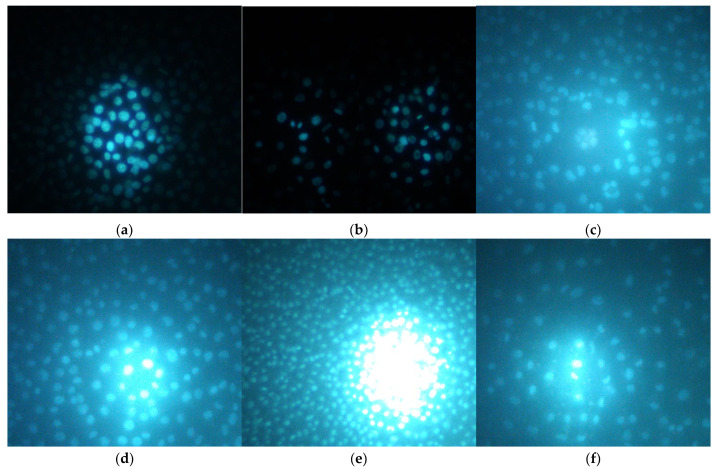
Hoechst staining of chosen samples after exposure to hypertonic buffer (2 h) or to *Hypericum* agents (48 h) with 100× and 200× magnification. The MIC values are for *S. aureus*. The panels are the following: (**a**) untreated control for the hypertonic buffer test; (**b**) hypertonic buffer; (**c**) untreated control for the *Hypericum* test; (**d**) RochC, dose of 2 × MIC; (**e**) olympiforin B, dose of 2 × MIC; (**f**) HirDM90, dose of 2 × IC_50_.

Hoechst is a dye that binds to DNA, hence making cell nuclei fluoresce blue. In the CCL-1 line, at 5 h after seeding, there was no monolayer yet. Mitotic spindles were dyed by Hoechst; therefore, active mitosis was noticed in all samples, and no cytopathic effect was observed (Figure S3). At 24 h, there was already a monolayer; mitosis was observed both in the untreated control and in all treated samples, including HirDM90 2 × IC_50_. Highly condensed and shrunken nuclei, a marker of apoptosis, were observed only when the cells were treated with hypertonic buffer before staining, but not in samples treated with daunorubicin or *Hypericum* agents at all time exposures (5 to 72 h) (Figure 9 and Figures S1–S6).

As a summary, there was no cytopathic effect from RochC and olympiforin B treatment with concentrations of MIC and 2 × MIC for *S. aureus* for all time points applied (Figure 8). Also, there were no signs of apoptosis—condensed nuclei and blebs—associated with any of the *Hypericum* agents at any of the tested time points. This is valid not only at low concentrations multiples of the MIC against *S. aureus* but also at high concentrations multiples of the IC_50_ (of HirDM90) (Figure 7, Figure 8 and Figure 9). The effect was antiproliferative, and further studies are needed to elucidate the underlying mechanism of action.

All the photographed images can be seen in Supplementary Figures S1–S6.

#### 2.3.7. Activation of Autophagy Was Detected with Western Blot

**Figure 10:**
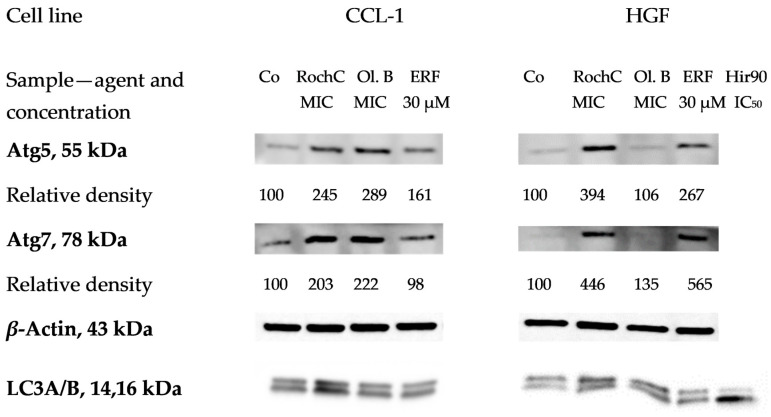
Expression of Atg5, Atg7 and LC3A/B proteins related to autophagy in CCL-1 and HGF cells treated with *Hypericum* agents. Legend: Co—untreated control, Ol. B—olympiforin B, Hir90—HirDM90. The MIC values are the concentrations for *S. aureus*.

Autophagy is an adaptive mechanism to stress factors such as starvation, cytotoxic agents, etc. Treatment with *Hypericum* agents induced (increased the expression of) proteins related to autophagosome formation (Figure 10). Atg5 and Atg7 were induced, indicating the activation of autophagy in all samples, while LC3A/B was converted to the lower migrating form visibly—also an indicator of autophagy—only in the positive control (30 µM erufosine) [59] and the HirDM90 IC_50_ treatment. Significant induction of Atg5 and Atg7 was observed. The proteins were increased two, three, and even four times (for RochC at HGF cells), even at the low MIC concentrations of the agents. Only olympiforin B led to a smaller increase in HGF cells (to 106–135%).

Further experiments may include antibodies for other proteins from the autophagic cascade, such as Beclin-1, Atg3, Atg16L1, etc.

#### 2.3.8. Cytochrome P450 (CYP450) Was Not Induced in HEPG2 Liver Cells with a Few Exceptions

CYP450 enzymes are responsible for the metabolism and inactivation of medications. The expression of CYP450 (quantity of protein) in HEPG2 liver cells was tested by enzyme-linked immunosorbent assay (ELISA). One of the main differences between the HEPG2 cell line and normal hepatocytes is the weak or absent expression of the cytochrome P450 (CYP) superfamily, e.g., CYP3A4, CYP1A2, etc. [60]. However, exactly that low basal activity of CYP proteins makes the cell line suitable for studies of CYP inducers [61].

**Figure 11:**
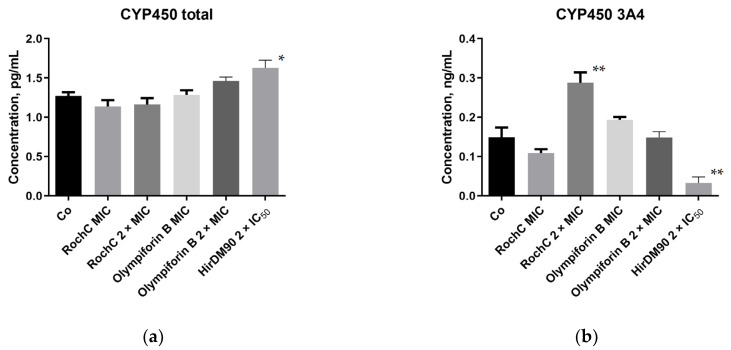
Effect of selected active substances from *Hypericum* on (**a**) human total cytochrome P450 (CYP450); (**b**) human CYP450 3A4 isoform in HEPG2 cells. The MIC values refer to *S. aureus*. The number of asterisks indicates the degree of significant difference according to One-way ANOVA (Tables S1 and S2): *—*p* ≤ 0.05; **—*p* ≤ 0.01.

It can be observed that at therapeutic concentrations appropriate for *S. aureus*, the selected *Hypericum* agents hardly induce the cytochrome system. Therefore, the risk that they would contribute to drug inactivation and the reduction in their bioavailability is low. This was valid for the total CYP450 and its main isoform, 3A4, which metabolizes approximately half of the drugs metabolized by CYP450 enzymes (Figure 11). The only exception was the dose of 2 × MIC of RochC, which induced CYP450 3A4. HirDM90 was deliberately applied at higher concentrations (2 × IC_50_), and it had a higher effect on both total CYP450 (inducing it) and 3A4 (inhibiting it).

#### 2.3.9. The Chosen Agents Did Not Irritate Rabbit Skin In Vivo at a Dose of Ten Times MIC

**Figure 12:**
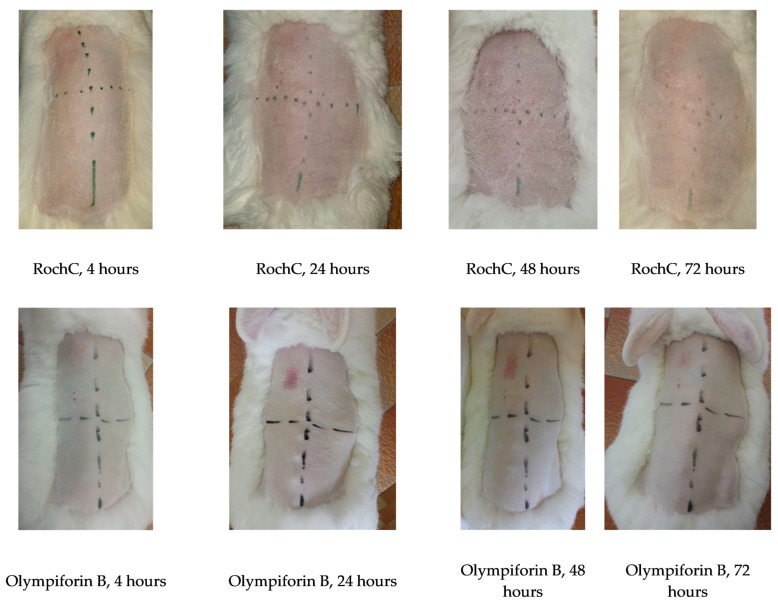
Lack of skin irritation on rabbits. The agents RochC and olympiforin B were applied at concentrations of MIC and 10 × MIC for *S. aureus*. Hours indicate exposure time. The positive SDS control is in the upper left panel, except for the last rabbit, where the exchange is indicated by arrows. One rabbit per agent, from a total of three rabbits per tested agent, is shown in the figure.

There was no skin irritation on rabbits, and the results indicate that RochC and olympiforin B do not cause skin toxicity at the MIC concentration for *S. aureus* and even at ten times the tested concentration (10 × MIC). Irritation was observed only in the positive control field with 10–20% SDS, in the upper left corner, as seen in Figure 12.

## 3. Discussion

### 3.1. Antibacterial Activity

In some cases, the naphtodianthrones [62], benzopyrans, xanthones [4], etc., in the *Hypericum* plants also exert an antibacterial effect. However, the compounds found in RochC were polyprenylated phloroglucinols, highlighting their strong antimicrobial action. They are typical of the *Hypericum* genus and found in other species. Olympiforin A has been found in *H. olympicum* [29]. Maculatoquiones A–D have been found in *H. maculatum* Crantz [63]. Hyperpolyphyllirin/hyperibine J has been found in *H. perforatum*, *H. androsaemum*, *H. tetrapterum* [64], *H. maculatum* [63], *H. triquetrifolium* Turra [65], *H. empetrifolium* Willd. [66], etc. HirDM90, an abundant fraction of an *H. hirsutum* extract that was used in higher quantities in the live-cell imaging and Western blot—in order to compare the effect of low and high doses of a cytostatic agent—had been found to contain other PPAPs such as hyperfirin or secohyperforin and adhyperfirin or adsecohyperforin [4].

Besides finding the most promising plants among those used in folk medicine, another main goal of calculating the total activity of a plant with the method of Eloff [6] is reversing, at least partially, the one-way benefit and flow of information. Scientists, with very little additional effort, could return to the local inhabitants of rural and less developed areas the favor of sharing ethnobotanical information about medicinal plants. *H. olympicum* and *H. hirsutum* are confirmed to be used in traditional medicine. The former is native to the Balkan Peninsula and northwestern Turkey and is used in Turkish folk medicine for stomachache, inflamed wounds and cuts [67]. *H. hirsutum* L., or Hairy St. John’s Wort, is a Eurasian herb with a large range from western Europe to western China [68]. It is used in traditional Asian medicine for hematochezia, irregular menstrual periods, and hematemesis [69]. There are unconfirmed data also for *H. rochelii*, or Rochel’s St. John’s Wort—with an area of the Balkan Peninsula [70]—that it is used in folk medicine to treat various ailments such as fever, headache, and stomachache, as well as an ornamental and fragrant plant [71]. Therefore, revealing that these plants have high total activity, i.e., strong antimicrobial activity and good exctractability, could be of help for the local communities where these species grow.

As discussed, biofilms of pathogenic bacteria may cause chronic infections and inflammation, which damage the surrounding tissues. Examples include chronic wounds, urinary tract infections, and endocarditis [38]. *P. aeruginosa* biofilms may lead to respiratory failure in cystic fibrosis patients [72]. Biofilms of foodborne pathogens in food production plants are also a threat to public health, as detached biofilm can spread infections and AMR to the environment, humans, and their microbiota [73].

In regard to staphylococcal biofilm on medical devices, such as catheters, mechanical heart valves, ventilators, and contact lenses, etc., the source can be the skin or other colonized body sites of patients or health care workers. Immunocompromised patients have an increased risk of developing infections when receiving a medical implant [41]. The polysaccharide adhesin (poly-N-acetylglucosamine polymer) in the staphylococcal biofilm is synthesized with the help of N-acetylglucosamine transferase, a product of the *ica*A gene. The product of *ica*D is required for maximum expression and optimal enzymatic activity of this transferase [74], which is associated with the formation of slime and biofilm and the phenotypic expression of capsular polysaccharide [75].

At 1/16 × MIC, Roch C induced *ica*A expression and decreased the *ica*D expression. This may be due to the stress factors in the media [2] or to the expression of *ica*R, the locus opposite of the icaABCD [76], as in the study with extracts from *Ginkgo biloba* L. [77]. In addition, there are data showing that with increasing glucose concentration, the biofilm increases, while the expression of the *ica*A and *ica*D genes decreases [78]; therefore, there is not always a straight correlation between the rise of the expression of the two genes and biofilm formation.

The constant mutations of MRSA may make the antibiotics that it is susceptible to soon ineffective [79], and new agents to combat this strain are needed. As mentioned, the direct bactericidal action of antimicrobials causes more AMR than the bacteriostatic action or the property of targeting some virulence factor, such as biofilm formation [44]. In addition, the MBIC and/or MBIC_50_ values being below the corresponding MIC values of an agent is beneficial. Low concentrations of the antimicrobial would achieve the aim of biofilm prevention, i.e., abolishing the phenotypic pathogenic expression, without influencing the microbial growth, including that of the normal human microbiota. Both active ingredients that this study focuses on, especially RochC, are promising for the mitigation of AMR in MRSA because of their sub-MIC MBIC_50_ concentrations for both tested bacteria and their sub-MIC MBIC concentrations for MRSA.

Considering the biofilm formation of *S. mutans*, St. John’s Wort extracts at a concentration of 20 µg/mL [80] and oil at a concentration of 512 μg/mL have inhibited it (including inhibition of *E. faecalis* biofilm) [81]. Given that the activity of RochC against *S. mutans* with an MIC of 6.25 µg/mL surpasses that of the St. John’s Wort oil with an MIC of 128 μg/mL, future investigation of the antibiofilm properties specifically against that bacterium deserves consideration.

### 3.2. Cytotoxic Activity on Normal Cell Lines

Research on the direct cytotoxic effect of active ingredients from *Hypericum* on normal and non-tumorigenic cell lines is a supporting component of broader studies focused on anticancer activity and thus does not always accompany such research. This is expected, as the aim to identify therapeutic potential is prioritized, and cytotoxicity in normal cells, although crucial, is often briefly addressed unless the agent shows promise. Limited funding resources often reserve such studies for follow-up investigations. If safe for normal cell lines in vitro, the final research step for a phytoconstituent would be mechanistic studies, animal models, and/or formulation development.

In the studies of the cytotoxic effect on both tumor and normal cells, the selectivity index (SI) is the ratio of the IC_50_ for a normal cell line to the IC_50_ for a cancer cell line. It is considered high and promising if it is a value greater than or equal to two, according to some authors [82,83], or greater than three, according to others [84]. Literature shows that the same extract can be more cytotoxic in vitro to a certain cancer cell line in comparison to a normal cell line, i.e., it can be a selective agent, but it can be less cytotoxic to another tumor cell line than to the normal type of cells [85].

A comparison between the IC_50_ values of the compounds in this study with their average IC_50_ values against tumor cell lines from another study [29] shows that olympiforin A is not very selective, as only its IC_50_ for HGF was higher than those for the tumor cell lines. Hyperpolyphyllirin/hyperibine J has a higher IC_50_ for HGF, CCL-1, and HEPG2. Olympiforin B is greatly selective, having a higher IC_50_ value for all cell lines in this study than its average IC_50_ for the tumor cell lines (1.3 µM), which further justifies its selection for additional experiments in the present work. The IC_50_ of the compounds and of other phloroglucinols from *H. olympicum* towards HEK-293 and the normal vascular endothelial cells EA.hy926 in the previous study varied from 0.9 to 34 µM. The SI for two normal cell lines varied between 0.35 and 7.14, meaning that the compounds in some cases were quite selective towards certain tumor cell lines.

The best selectivity, according to our knowledge, is so far demonstrated by a methanol extract of *H. hookerianum* Wight ex Arn., with an SI reaching 10–50 for tumor lines with respect to the normal monkey Vero kidney epithelial cell line. The IC_50_ values against Vero were between 100 and 271 µg/mL [45].

The methanol extract and its fractions, as well as the xanthones and phloroglucinols isolated from *H. roeperianum* Schimp. ex A.Rich, had IC_50_ values against AML12 normal hepatocytes in the range of 42 to >80 µg/mL and 21 to >165 µM, respectively. They also possessed very good SI within the range of 1.2 to 4.6 and 0.9 to >3.74, respectively [24].

There are cases where the pro-apoptotic action of a PPAP, e.g., HF, is selectively observed in cancer cells, rather than in healthy cells. For instance, the same concentration of HPF that induces distinct damage in B leukemic cells does not harm the viability of human B lymphocytes from healthy donors at all [86]. The current hypothesis explaining this is related to the fact that HPF is a protonophore. In both tumor and normal cells, the negative cytosolic side of the plasma membrane facilitates proton influx, but in cancer cell lines, the pH gradient (∆pH) across the plasma membrane is greater because of the higher activity of proton channels that extrude protons from the cells. Thus, in the presence of a protonophore, only a faint proton entry is made in normal cells, but a significant and persistent cytosolic proton influx occurs in cancer cells, restoring cytosolic acidity and allowing apoptosis. At the same time, HF makes the extracellular tumor microenvironment less acidic, thus impeding tumor cell migration and invasiveness and extracellular matrix digestion. In addition, cancer cells have a hyperpolarized inner mitochondria membrane and a higher concentration of HF could collapse this membrane, thus leading to cell death.

This hypothesis is yet to be verified in cancer cells but is already confirmed by Sell et al. [87] in both the plasma membranes of normal cells and a synthetic lipid bilayer without channel proteins. HF caused proton entry into the cell and cytosol acidification directly dependent on the proton gradient between the two sides of the membrane. Interestingly, the authors also demonstrated that HF accumulates in the membrane, which might explain the effects of even low doses of HF and the extracts containing it. Other PPAPs from *Hypericum*, such as olympiforins A and B, being also lipophilic and protonophores, likely have the same mechanism of action [88].

An *H. perforatum* extract had an IC_50_ against seven tumor cell lines in the interval of 6.7 to 45 µg/mL. The IC_50_ for the healthy human lung fibroblasts CCD-34Lu was 13.6 µg/mL, and the IC_50_ for HEK293 cells was higher than in the current study—28.3 µg/mL [89]. The IC_50_ values of an olive oil extract from that plant were high and not very different for SW-480 and bone marrow-derived mesenchymal stem cells—4800 and 4900 mg/mL, respectively. The cell migration and colony formation were significantly reduced at the IC_50_ values for both cell lines [90]. Different organic solvent extracts from *H. perforatum* aerial parts had an average IC_50_ of 28 µg/mL on three cancer cell lines and an average IC_50_ of 24 µg/mL on the normal fibroblasts MRC-5 [85]. The cell viability of the non-tumorigenic brain endothelial cell line hCMEC/D3—a blood–brain barrier model—was moderately affected by an *H*. *perforatum* decoction with an IC_50_ of 732 μg/mL, but NSC-34—a hybrid cell line of neuroblastoma and mouse motoneurons—was even less affected with an IC_50_ of >1000 μg/mL [91]. The effect of a methanol extract from that species on PC-3 human prostate cancer cells and the normal human chondrocyte cell line C28/I2 was compared. Upon 48 h treatment at 2.720 mg/mL (about twice the dose of IC_50_), 82% of PC-3 cells underwent death, while C28/I2 cells remained viable up to 65% under similar conditions. The CD82 protein has the function of inhibiting tumor metastasis and thus is a therapeutic target in prostate cancer cells. A two-fold increase in the relative gene expression of CD82 in PC-3 cells was revealed in comparison to the untreated control. The increase in the normal cells was much less impressive, suggesting again a selective action of the plant only upon malignant tissues [46]. *H. perforatum* extract encapsulated in poly(lactic-co-glycolic acid) nanoparticles for 24 h at a dose of 5 mg/mL inhibited KYSE30 cancer cells by approximately 70% and normal squamous cells by up to only 29%. Cyclin D1 is a therapeutic target for esophageal cancer, and the nanoparticles exerted selectivity, causing a significant decrease in cyclin D1 expression in the KYSE30 cells, while in the normal cells it was at least 2-fold higher [92].

Although the IC_50_ for a breast cancer cell line of hypericin was significantly lower when compared to cisplatin—5 vs. 20 μg/mL for 24 h—the two compounds did not have a significant effect on the cell survival of unspecified fibroblasts at concentrations up to 30 μg/mL for 24 h [93]. Hypericin is a naphtodianthrone and another major secondary metabolite of *H. perforatum*. It has a phototoxic effect since it is able to produce reactive oxygen species (ROS) as a result of adequate photoexcitation, so numerous works have revealed that it has no cytotoxicity in the dark. It also accumulates much more in neoplastic tissue than in normal tissue [94], and likely for that reason, it induced apoptosis in a gastric cancer cell line but not in normal human fibroblasts [95].

The aqueous and organic solvent extracts from *H. empetrifolium* and *H. lydium* Boiss. had IC_50_ values against HEK-293 cells varying from 63 to 304 μg/mL. Unfortunately, they were more cytotoxic to HEK-293 cells than to the three tumor cell lines DU-145, A549, and MCF-7. The only exception was an acetone/water extract from *H. empetrifolium* with an IC_50_ of 189 μg/mL against DU-145 prostate cancer cells and 304 μg/mL against HEK-293 [47]. None of the phenolic substances (flavonoids, acids, etc.) isolated from *H. cerastioides* (Spach) N.Robson had toxicity to a normal fibroblast cell line, L929 (IC_50_ > 200 µM). Cerastioside A, a normonoterpene, and I3-II8-biapigenin displayed selectivity, as they had weak cytotoxic activity against a panel of cancer cell lines with IC_50_ values in the interval 107–198 μM [48]. The ethanol extracts prepared in the beginning of flowering and full flowering periods from *H. heterophyllum* Vent. again did not have a significant cytotoxic effect in L929 cells, while being cytotoxic to MDA-MB-231 breast cancer cells [20].

One of the few works comparing the antimicrobial effect and the toxicity on normal cell lines of *Hypericum* ingredients [96] reported that methanol, petroleum ether, and ethyl acetate extracts from *H. triquetrifolium* and *H. scabrum* L. aerial parts had an antimicrobial effect, with MICs against *S. aureus* and *S. epidermidis* between 90 and 5000 µg/mL. The extracts did not show toxicity in a normal cell line (L-929) at a concentration of 100 µg/mL (cell viability over 70% and up to 91%, no exposure time mentioned). In another study, the methanol leaf extract of *H. triquetrifolium* had an average IC_50_ for four cancer cell lines of 142 μg/mL. The average IC_50_ (*n* = 4) for the normal cell line WRL-68 (HeLa derivative) was 314 μg/mL (24 h exposure for all cell lines). Therefore, the SI was over two, and the extract was selective. In addition, the extract did not have any cytogenetic effect, because it raised the metaphase index of the bone marrow cells in healthy mice from 5.2% to 5.5, 6.7, and 7.8% at 50, 100, and 200 mg/kg, respectively, while cyclophosphamide reduced it to 3.6% [97].

An ethyl acetate extract from *H*. *japonicum* was more cytotoxic to human lung epithelial tumor cells (A549) than to normal human lung fibroblast cells (WI-26VA4) [98]. Balikci et al. found the major components of the aerial parts of a methanol extract from *H. olympicum* to be volatile compounds such as eicosane, heptacosane, 2-propen-1-ol, etc. This extract had an IC_50_ against breast cancer cell lines of approximately 25–40 μg/mL. In human lymphocytes, the concentrations up to 1750 µg/mL induced genotoxic activity without decreasing the mitotic index. The extract caused significant DNA damage at selected doses (250–750 μg/mL), as shown by a comet assay, while chromosomal damage, i.e., genotoxic effect, was observed at relatively high doses (≥500 µg/mL) by employing sister chromatid exchange and micronucleus methods. The extract did not act on the mechanisms pertaining to the proliferation of the cells [99]. Fifteen substances from *H. longistylum* Oliv. (sesquiterpenes and flavanones) did not harm normal mouse lung fibroblasts [100].

An interesting study sought potential side effects of *H. connatum* Lam. and *H. caprifoliatum* Boiss. on the placental development and function. The plants could be used as drug alternatives to mild depression or viral infections by pregnant women. The non-fusogenic JEG-3 cells and the fusogenic BeWo cells were used. They both originate from tumor cell lines, but are used as an in vitro model for studies of the normal placental trophoblast. The differentiation of this trophoblast yields the syncytiotrophoblast cell, whose successful formation and expansion are crucial for the functioning of the placenta. The main functions of syncytiotrophoblasts are absorption, exchanges, specific hormonal secretion, and fetal Ca^2+^ homeostasis regulation. The studies on the viability of the trophoblast-like cells showed that the methanol extract of both plants, as well as the hexane extract from *H. connatum* (HCo), did not harm the BeWo cells up to 30 μg/mL. However, these extracts were cytotoxic to JEG-3 cells, as was the hexane extract from *H. caprifoliatum* (HCa) to the BeWo cells. In doses as low as 5 μg/mL, they significantly decreased the cell viability. Still, in the case of 5 and 15 μg/mL of HCa, cells were morphologically unchanged as observed by microscopy. All concentrations and plant extracts exerted a significant decrease in the biochemical cell differentiation (hormone production). However, the inhibition of the morphological cell differentiation (cell fusion) was significant only for the HCa at 15 μg/mL. In addition, *H. connatum* was revealed to interfere with the Ca^2+^ transport system. Both methanol extracts contained phenolic acid and flavonoids. The hexane extracts from both plants presented dimeric acylphloroglucinols, and there was a tautomeric mixture of unresolved acylphloroglucinols in the HCa. The results indicated that the two *Hypericum* species extracts can interfere with trophoblast viability, differentiation, and Ca^2+^ influx. Their intake by pregnant women should be cautious, although more in vivo research is necessary for assessing the full extent of their effect [101].

The phototoxic effect of the ethanolic extracts of eleven *Hypericum* species was studied on NIH/3T3 normal murine fibroblasts. The IC_50_ values in the dark (light-independent cytotoxicity) were between 101 and 267 µg/mL. Those values under light were 31–256 µg/mL. The ratio of two (a higher ratio corresponds to higher photosensitizing and phototoxic activity) varied from 0.9 to 4.3. As expected, under light exposure the light-dependent cytotoxicity was higher for those species with a higher content of naphthodianthrones. The lowest cytotoxicity, under both dark and light conditions, was observed for *H. hirsutum*, which lacked hypericins. The high toxicity of that species in the current study highlights that fractions of less polar solvents could have different activity. The three most active species, *H. perfoliatum* L., *H. perforatum*, and *H. tetrapterum*, had the highest ratio of dark:light toxicity and high amounts not only of naphthodianthrones but also of HF [25].

While the direct cytotoxic effect on normal cell lines has not been often elucidated, *Hypericum* constituents are reported to have in vivo wound healing properties, possibly attributed to their anti-inflammatory, antimicrobial, and antioxidant activity. For example, oily extract of *H. perforatum* was found to effectively reduce scar heights in human tissue [102].

Hong et al. [103] reported that *H. hookerianum* ingredients strongly protect HT-22 murine hippocampal cells from glutamate-induced cell death and SH-SY5Y cells from 6-hydroxydopamine (6-OHDA)-mediated neurotoxicity. Cell viability was reduced to approximately 50% after exposure to 5 mM glutamate and to 36% after exposure to 6-OHDA. The plant ingredients, e.g., the dichloromethane and ethyl acetate fractions with concentrations of 6, 17, and 50 μg/mL, were able to restore the viability almost completely. The effective concentration 50 (EC_50_)—in this case, the concentration where the viability was restored to about 75%—of 4-hydroxy-2,6,4′-trimethoxydihydrochalcone and sesamine, out of several compounds isolated from that plant, was 1.48 µM and 2.85 μM, respectively. Therefore, extracts and compounds from *H. hookerianum* are capable of significant neuroprotective effects in HT-22 and SH-SY5Y cells. Only the n-butanol fraction at 50 μg/mL showed cytotoxicity.

*Hypericum* ingredients have also been frequently reported to have protective and anti-inflammatory activity in non-tumorigenic cell lines, tissues, and animal models of acute and chronic inflammation.

For example, HF and an HF-containing *H. perforatum* extract both markedly inhibited interferon-elicited signaling pathways in pancreatic beta cells and in rat and human pancreatic islets, leading to prevention of inducible nitric oxide synthase (iNOS) gene expression and protection against cell damage. HF also influenced the pro-inflammatory and immunological responses of mouse microglia and macrophages which are involved in the progression of neuropathological disorders [104]. Pretreatment with *H. perforatum* extract protected the PC12 cell line—an immortalized cell line derived from a rat pheochromocytoma—from H_2_O_2_-induced ROS generation and damage in a concentration-dependent manner (1–100 µg/mL) [105]. In zymosan-injected mice, pretreatment with *H. perforatum* extract led to an increase in the intracellular amounts of antioxidant enzymes associated with the reduction in ROS levels and iNOS expression and to a decrease in the interleukin 1β production, as compared to untreated controls [106]. In summary, Menegazzi et al. give a lot of instances in their review [88] that HF-containing *H. perforatum* extract or HF can attenuate inflammatory response and subsequent tissue injury triggered by injuring stimuli in several cell types and animal models, mainly by lowering ROS production and downregulating the expression or activity of inflammatory mediators.

In regard to the results of the current study, all the tested agents had cytotoxicity comparable to clinically used drugs such as cisplatin [85,107,108,109]. They were least toxic to HGF. For comparison, the other *Hypericum* agent besides RochC that has presented an antibacterial MIC value of only 0.625 µg/mL, a hydroalcoholic extract of *H. perforatum* containing 0.1 mg/mL hypericin, had an IC_50_ value of only 0.604 μg/mL against the human gingival fibroblasts HGF1-PI1 [34]. The IC_50_ of RochC against HGF in the current study is 9.22 μg/mL, i.e., it is much more selective and spares the gingival cells.

It is noteworthy that 1 µM of the PPAP HF was found to trigger differentiation in primary cultures of human keratinocytes and in derived HaCaT cell lines and to inhibit their proliferation [110]. If we compare our results, where the IC_50_ of the PPAPs used in this study was between 2.7 and 4.7 µM, it appears that a small dose can have a differentiating effect, while a higher dose can exert cytotoxic activity on skin fibroblasts.

A literature inquiry showed that the CFU assay from ISO 10993-5 has not been applied to HEPG2 cells before, to the best of our knowledge. Nevertheless, CFU tests with different protocols have been applied to this line [111,112], including one with a semi-solid medium and a seeding concentration similar to ours [113].

However, that protocol did not include an approximate counting of the cells in a colony in order to start the detection of results after they reach 20–50 cells per colony. In our case, HEPG2, after reaching about 20 cells in a colony, formed a kind of plaque covered with a substance similar to fat droplets, and in this aggregate, individual cells could not be distinguished and counted (Figure S8a). Generally, this cell line yielded poor results for the CFU assay in our case, with treatment dramatically reducing their clonogenic potential and/or them being poorly clonogenic in the untreated control in the first place.

For fibroblast cells, such as HGF and CCL-1, a different protocol could be used, named CFU assay for fibroblasts (CFU-F), but it demands different reagents [114,115], so we tried the CFU assay from ISO 10993-5 as for the other cell lines. Unfortunately, HGF did not form colonies but a network (Figure S8b,c).

Some synthetic or natural cytotoxic agents produce distinct features of apoptosis, necrosis, or other types of cell death in the cell. For example, Hoechst staining after podophyllotoxin exposure produced clear nuclear condensation and shrinkage in HaCaT cells, a sign of apoptosis [116]. However, cytotoxic agents may not directly kill cells but inhibit their division and multiplication, i.e., be cytostatic. In the current study, not only RochC and olympiforin B in the low MIC doses, but the undoubtedly cytotoxic doses of 2 × IC_50_ of HirDM90 did not cause any nuclear and DNA condensation, the distinct signs of apoptosis, even after 24–48 h exposure in the few surviving cells. The concentration of 2 × IC_50_ of HirDM90 certainly resulted in a much less dense layer of cells and fewer cells, but their nuclei appeared normal and intact. Therefore, the tested *Hypericum* agents are most likely cytostatic. Nevertheless, olympiforin A [29], HF [117] and other PPAPs [108] have been reported to either induce apoptosis or activate caspase 9 in tumor cell lines. We know that the *Hypericum* ingredients are selective and target and kill mostly cancer cells. Therefore, it is worth elucidating the mechanism of action for their cytotoxic effect on normal cells when applied in high enough concentrations.

Autophagy is a process of autophagosomal–lysosomal degradation of cytoplasmic components. This process is activated by nutrient deficiency, infection, or other stress factors and is associated with neurodegenerative and other diseases but is also observed in physiological processes such as development, differentiation, etc. Autophagy is an adaptive and pro-survival mechanism of the cell to metabolize its components that are not vital and immediately important for survival. If starvation or other stress factors continue, autophagy is not sufficient as a compensation, and excessive autophagy may lead to cell death, a process morphologically distinct from apoptosis [118].

The Atg genes control autophagosome formation through Atg12-Atg5 and LC3B (Atg8-II) complexes [119]. During autophagy, LC3A is converted to LC3B through lipidation by a ubiquitin-like system involving Atg7 and Atg3 that allows LC3B (the lipidated form of LC3) to become associated with autophagic vesicles and attached to the autophagosome membrane. The presence of LC3 in autophagosomes and the conversion of LC3 to the lower migrating form, LC3B, are used as indicators of autophagy [120].

The results of this study showed that Atg5 and Atg7 were induced significantly in most samples, indicating the activation of autophagy, while LC3A/B was converted to the lower migrating form visibly—also an indicator of autophagy—only in the positive control (30 µM erufosine) and the HirDM90 IC_50_ treatment. Further experiments may include antibodies for other proteins from the autophagic cascade, such as Beclin-1, Atg3, Atg16L1, etc.

### 3.3. Effect on Other Factors of the Host Homeostasis—CYP450 and Beneficial Lactobacilli

*H. perforatum* is a notorious CYP450 inducer, which reduces the efficacy of drugs such as digoxin, indinavir, warfarin, oral contraceptives, etc. The degree of CYP3A4 induction correlates significantly with the content of HF, a PPAP [121]. Testing for effects on hepatic enzyme systems’ activity of the PPAP olympiforin B and the extract RochC rich in PPAPs—agents selected for their optimal cytotoxic profile—showed that at concentrations inhibiting *S. aureus*, they hardly induce total CYP450 and its main isoform 3A4. The only exception was the dose of 2 × MIC of RochC, which induced CYP450 3A4, and this has to be taken into account for possible future application.

As mentioned, the data on the direct in vitro effects of *Hypericum* on beneficial lactobacilli is limited and a niche field. However, lactobacilli can have a dual role. For instance, *L. plantarum* has a beneficial and probiotic role when in fermented foods, but on our teeth, although a part of the normal oral microbiota and not a cavity initiator like *S. mutans*, it can be a secondary caries invader [80]. *Lactobacillus acidophilus* is an essential member of the natural vaginal microbiota but can also be cariogenic. Thus, the research is mainly focused on oral lactobacilli involved in tooth decay. For example, an *H. perforatum* extract had an MIC value over 300 μg/mL against *L. acidophilus* [122]. The aqueous fraction of an ethanolic extract of *H. perforatum* had a MIC of 8 μg/mL against *L. plantarum*, and its alcoholic extract suppressed *L. acidophilus*, thus allowing us to consider the plant for potential oral disinfectant formulations [34,80].

In regard to the beneficial role of lactobacilli, topical preparations of *H. perforatum* oil did not affect *Lactobacillus acidophilus*, therefore highlighting the possible selectivity of such formulations [123], since they inhibited some pathogenic bacteria such as *S. pyogenes*, *Moraxella catarrhalis*, etc. The study suggests that application of the ointments will not distort the normal vaginal microbiota. Milutinović et al. examined the effect of two extracts of *H. perforatum* on probiotic *L. rhamnosus* and *L. plantarum*. The first extract was made according to the standard procedures of the European Medicines Agency (EMA) with 50% ethanol, and the second one was an ethanol extract produced under optimized microwave-assisted extraction (MAE). The EMA extract inhibited the growth of two *L. rhamnosus* strains with MICs of 10 and 20 mg/mL and did not suppress *L. plantarum*. The MAE extract did not suppress *L. rhamnosus* and stimulated the growth of *L. plantarum* [124].

There is also interesting research focusing on the in vivo administration of *H. perforatum* in animals and following the changes in their gut lactobacilli and other microbiota members. The *Lactobacillus* population increased significantly, and the *Escherichia coli* population decreased in the gut of broilers whose drinking water had been supplemented with 150 to 250 mg/L of a hydroalcoholic extract from the plant [125]. In broilers again, the tested olive oil extract at doses of 3 to 4.5 mL/kg increased the total lactic acid bacteria count and decreased the total *Enterococcus* spp. counts. Interestingly, the 1.5 mL/kg dose and powdered *H. perforatum* added to the basal diet decreased the total lactic acid bacteria count [126]. A St. John’s Wort extract significantly elevated the abundance of *Lactobacillus* and other bacterial genera in the rat gut, which could contribute to the attenuation of hypercholesterolemia indirectly by modulating metabolic pathways in the host–microbiota system [127]. This could be related to the fact that the antidepressant effect of St. John’s Wort seems to be partly connected to the restoration of gut microbial composition by enriching the *Akkermansia muciniphila* intestinal symbiont bacterium, which leads to a reduction of microbiota-derived kynurenine levels, an increase in 5-hydroxytryptophan levels, and regulation of the NFκB-NLRP2-Caspase1-IL1β pathway [10].

Since *Hypericum* plants are rich in polyphenols, it is important to notice that they and their metabolites have a positive impact on the gut microbiota. They increase the levels of *Lactobacillus* and *Bifidobacterium* both in vitro [128] and in vivo. Regular consumption of polyphenol-rich foods increases the levels of these probiotic bacteria while decreasing pathogens such as *Clostridium*, *S. aureus*, etc. [129]. In addition, the effect of polyphenols on the human host is typically mediated through interaction with the gut microbiota because of their poor absorption in the digestive tract. The microorganisms catabolize them to metabolites with antioxidant and beneficial effects that could be transported within the host and take part in preventing chronic diseases such as cancer, diabetes, etc. Or the catabolites could in turn affect the gut microbiota, thus contributing to health promotion, for example, through the intestinal immune function [130]. For that reason, polyphenols can be named a novel group of prebiotics [129,131].

All the contrasting findings underline the importance of evaluating the specific effects of *Hypericum* active ingredients on beneficial lactobacilli when considering therapeutic applications.

For the purposes of this work, Lactobacillaceae species from the list of the European Food Safety Authority (EFSA) with qualified presumption of safety (QPS) or generally recognized as safe (GRAS) status (according to the Food and Drug Administration (FDA)) were selected. As part of a laboratory collection, they were characterized as candidate probiotics, fulfilling EFSA’s criteria [132]. Thus, selected *L. fermentum* from human origin and *L. plantarum* from dairy origin were included. In the current study, the MIC values for the tested candidate-probiotic strains were higher than those obtained for *S. aureus*, implying that both agents targeted the pathogen and could be used at anti-staphylococcal doses without significantly compromising beneficial lactic acid bacteria (LAB).

Many antibiotics used against staphylococci, especially if they have broad-spectrum Gram-positive activity, such as clindamycin [133] or vancomycin [134], are also active against Gram-positive LAB, which may lead to overgrowth of opportunistic *Candida* and other pathogens as well as gastrointestinal disturbances [50]. This is consistent with the successful in vitro experiments for the use of prebiotics and probiotics to prevent infection [135].

The higher MICs against candidate probiotic lactobacilli of RochC and Olympiforin B reinforce their safety and selectivity, important features for topical or systemic anti-staphylococcal treatments. Whether they would be less affecting and more unlikely to harm the normal microbiota than broad-spectrum Gram-positive antimicrobials is a question for future research.

There are observations of market trends suggesting that plant extracts and possibly their compounds can be used as food additives in flavored fermented dairy products containing lactic acid bacteria [136]. Further in vivo research to establish the effect of *Hypericum* phenolics specifically on the complex host–microbiota–pathogens system is needed. This is to be considered for possible applications and formulations of these phenolics, such as PPAPs, flavonoids, phenolic acids, hypericins, etc., in the direction of functional foods, rather than pharmaceuticals.

### 3.4. Limitations of the Study

Undoubtedly, the main limitation of the study is that it is in vitro research, which does not include an in vivo infection model. Indeed, it includes cytotoxicity tests on normal mammalian cell lines, but they are a supplement and not yet a replacement when developing an antimicrobial candidate drug. This limitation is not valid for the lactic acid bacteria, because according to the criteria of EFSA, in vitro tests on commercial or candidate probiotic strains are sufficient [132].

In the present, the pathway before human clinical trials must include an infection model, for instance, a model of biofilm on a wound, dental caries, skin, soft tissue infection, pneumonia, etc. The requirements for even superficial infections, such as cutaneous or dental cavities, often include the sacrifice of the test animals for histopathological examination. Although in vitro tests have their limitations in predicting the pharmacokinetics, host immune interactions, and tissue penetration of the pre-drug, they are mandatory to be pioneering at the preclinical stage. Therefore, from an ethical point of view, the authors find that the main limitation of the work may be considered an advantage.

The limitation of the lack of systemic toxicity elucidation of the agents can be partly overcome by in silico tests, cytotoxic tests on 3D cell culture models and organ-on-chip systems, which are one step further than 2D cell cultures and are a good plan for future research.

The MTT assay and live-cell imaging performed in the current study have their limitations of being unable to determine the mechanism of the cytotoxic and antiproliferative effect of PPAPs and PPAP-rich extracts on normal cell lines when in high enough concentrations. The low number of antibodies for autophagy detection tested is another limitation of the work. Last but not least, the limited quantity of compounds, e.g., hyperpolyphyllirin/hyperibine J, due to the unpredictable yield of plant samples, is another limitation of the study, and overcoming it would need time in order to wait for the new flowering season. The listed limitations warrant further studies.

## 4. Materials and Methods

### 4.1. MIC and MBC Evaluation

The MIC and MBC assessment on the pathogenic bacteria and the candidate probiotic lactic acid bacteria was carried out by the broth microdilution assay (BMD) and the agar plate assay, respectively, as described in [4]. The metabolic (dehydrogenase) activity of the lactic acid bacteria was assessed as described in [4].

#### 4.1.1. MIC and MBC Evaluation on Pathogenic Bacteria

*P. aeruginosa* PAO1 ATCC 15692 was purchased from the American Type Cell Culture Collection (ATCC, Manassas, VA, USA). *S. mutans* DSM 20523 was bought from the German Collection of Microorganisms and Cell Cultures (DSMZ GmbH, Braunschweig, Germany). All other bacteria and strains were purchased as described in [4].

#### 4.1.2. MIC Evaluation on Lactobacilli

Lactobacilli of different origins from the collection of Laboratory “Lactic Acid Bacteria and Probiotics”, the Stephan Angeloff Institute of Microbiology, Bulgarian Academy of Sciences (Sofia, Bulgaria), were selected (Table 13). The strains had been assessed previously, as candidate probiotics, according to the in vitro criteria recommended by the EFSA and the WHO guidelines [132]. They were stored at −20 °C in MRS broth (Merck Group, Darmstadt, Germany) supplemented with 20% v/v sterile glycerol. Prior to the experiments, lactobacilli were subcultured twice in MRS broth (GM369, HiMedia, Mumbai, India) at 37 °C. An overnight exponential-phase culture (5% *v*/*v* inoculum) was used for the tests.

### 4.2. Time Kill Assay

A time kill assay was performed according to the modified protocol of Sendi and Ruppen, 2015 [137]. A bacterial suspension of *S. aureus* was treated with two concentrations of the selected test agents (MBC and 1/2 MBC), as in the broth microdilution assay, and three decimal dilutions were immediately made from it. From these dilutions, 25 µL was seeded in brain heart infusion agar in a well of a 12-well plate. An untreated control was plated in the same way. Then, the bacterial suspension (treated and untreated) was placed in an incubator (MLW Kombinat, Leipzig, Germany) at 37 °C. Again, 25 µL of it was taken for seeding after 1, 2, 3, 4, 6, and 24 h, which equals a total of 7 time points. After 16–24 h of incubation, the colonies in the seeded plates from all dilutions were counted, and the number of colony-forming units (CFU) per mL, or in other words, bacteria per mL, was calculated.

### 4.3. Biofilm Assay

The biofilm assay was performed according to the protocol of Stepanović [138], as already described [4].

### 4.4. MTT Assay for Cytotoxicity

The MTT assay was performed according to Annex C, ISO 10993-5 [56] as in [116]. The panel of non-tumorigenic cell lines consisted of one murine (CCL-1 fibroblasts, which are recommended as a standard for cytotoxicity evaluation in Annex C of ISO 10993-5 [56]) and four human cell lines—HaCaT (immortalized epidermal keratinocytes), HGF (gingival fibroblasts), HEK-293 (embryonic cells from kidney epithelium), and HEPG2 (hepatoblastoma). Although technically a cancer cell line, HEPG2 is non-tumorigenic and retains many hepatocyte-related features in its proteome and a high degree of morphological and functional differentiation, also being a stage one hepatoblastoma [139,140,141,142,143].

CCL-1 was purchased from ATCC and HaCaT and HGF were bought from CLS Cell Lines Service (GmbH, Eppelheim, Germany). HEK-293 and HEPG2 were obtained from DSMZ. The cell culture medium for HaCaT and HEPG2 was Dulbecco’s Modified Eagle Medium (DMEM) with 4.5 g/L glucose (DMEM-HA). For HGF it was DMEM/F-12 (DMEM-12-A, a 1:1 mixture of DMEM and Ham’s F-12 medium). The cell medium for CCL-1 and HEK-293 was Eagle’s Minimum Essential Medium (MEM) (MEM-A). Ten percent heat-inactivated fetal bovine serum (FBS) was added to all media but to the one for the CCL-1 cells, to which 10% heat-inactivated horse serum was added. All media were supplemented with 2 mM L-glutamine and Penicillin/Streptomycin (Pen/Strep, 100×). The cell culture media, sera, and additives were from Capricorn Scientific (Düsseldorf, Germany).

### 4.5. Colony-Forming Unit Assay

The CFU was also performed according to ISO 10993-5 [56]. Briefly, the cell lines were seeded at a concentration like for the MTT assay in 6-well plates, treated with the chosen agents and left for a 48 h exposure. Then, the cells were trypsinized, and pre-calculated 20 to 100 µL from the cell suspension was added to a sterile Roswell Park Memorial Institute (RPMI) medium (RPMI-A, Capricorn Scientific), containing 20% of the respective cell culture medium for each cell line but with a final concentration of FBS and L-glutamine of 40–42% and 0.8–1.2 mM, respectively, 0.2% NaHCO_3_ (S6297, Merck Sigma-Aldrich, Saint Louis, MI, USA), and 0.8% methylcellulose (M0512, Merck Sigma-Aldrich) for viscosity. The viscous cell solution was vortexed for 10 s and dispensed in an equal number of cells per well in 6-well plates (1.2 mL per well) in triplicate for each concentration of treatment. HaCaT and HEK-293 were seeded at a concentration of 2500 cells per mL, CCL-1 and HEPG2—2000 cells per mL, and HGF—1000 cells per mL. The plates were incubated for 6 to 10 days, and when the colonies reached 20 to 50 cells per colony, 100 µL MTT salt solution was added to each well. After half an hour, the stained colonies were counted manually using a microscope.

### 4.6. Gene Expression of Adhesion Genes

#### 4.6.1. Isolation of RNA and cDNA Synthesis

The preparation of the samples for the RNA isolation was carried out as for the biofilm assay. RNA from the treated samples and the untreated positive MRSA control was isolated with the help of the Environmental DNA & RNA Purification Kit (E3572, EURx Ltd., Gdańsk, Poland). The chosen treating concentrations were sub-MICs—1/8 × to 1/32 × MIC, or 1.225, 0.613, and 0.306 for RochC and 0.125, 0.0625, and 0.031 µg/mL for olympiforin B. The protocol of the manufacturer was followed with some optimization. A total of 50 µL lysozyme (20 mg/mL) from chicken egg white (L6876, Merck Sigma-Aldrich) and 10 µL lysostaphin (L4402, Merck Sigma-Aldrich) from *Staphylococcus staphylolyticus* (200 U/mL) were added per sample. The isolated quantity of RNA was measured on a NanoDrop Lite Spectrophotometer (Thermo Scientific, Waltham, MA, USA) and standardized to equal concentrations of RNA (1 μg in 20 μL) according to the protocol of iScript Select cDNA Synthesis Kit (Bio-Rad Laboratories, Inc., 170-8896, Hercules, CA, USA) for reverse-transcription PCR (RT-PCR) on C1000 TouchTM PCR Thermal Cycler (Bio-Rad Laboratories, Inc., Singapore, Southeast Asia). The samples, being bacterial, were subjected to cDNA synthesis using random hexamer primers and reverse transcriptase enzymes. The obtained cDNA samples were stored at −80 °C and used for real-time PCR.

#### 4.6.2. Real-Time PCR

Quantitative real-time PCR was prepared using Sso Advanced Universal SYBR Green Supermix (Bio-Rad Bio-Rad Laboratories, Inc., 1725270, Hercules, CA, USA) on a CFX96 TouchTM Real-Time Detection System (Bio-Rad, Singapore, Southeast Asia) in order to determine the expression of the *ica*A and *ica*D genes. The *rho* gene (coding a gene for transcription termination factor [144]) was used for reference and normalization (Table 14).

#### 4.6.3. Gene Expression Analysis

The quantity of gene expression of the *ica*A and *ica*D expression levels in MRSA was determined by the 2^−∆∆Ct^ method [145]. For normalization of the data, the *rho* gene was used as a housekeeping gene.

### 4.7. Live-Cell Imaging for Cytopathic Effect in Unstained Cells and Cells Stained with Hoechst for Apoptosis Detection

The cytopathic effect assay was performed in 24-well plates using the CCL-1 cell line, as recommended in ISO 10993-5 [56]. The seeding concentration was the same as in the MTT assay. Treated cells were viewed under an inverted microscope without fluorescence filters and documented microscopically.

The Hoechst 33342 dye (B2261, Merck & Co., Inc., Rahway, NJ, USA) was used to monitor apoptosis in the CCL-1 cell line, too. Cell suspension was seeded in the same manner as for MTT, but 50 µL per well in 96-well clear bottom plates. Overnight culture was treated with the *Hypericum* agents, and after the defined exposure time, the recommended amount of dyes was added, and after 20–30 min in the dark, the cells were observed under an inverted fluorescence microscope (BOE 5000.930, mode BIB100, BOECO, Hamburg, Germany) and photodocumented microscopically (B-CAM16, BOE 1900.16000, BOECO, Hamburg, Germany) with the application software B-View (BOECO, version x64, 4.7.14531.20190425). Daunorubicin (30450, Merck Sigma-Aldrich) and hypertonic buffer were used as positive controls, and the treatment protocol and recipe of the latter were taken and adapted from the kit Cell Death Detection ELISA (11544675001, Roche, Basel, Switzerland). For the hypertonic buffer, the exposure time was 2 h, and the cells were plated at twice the concentration as for the MTT and cytopathic tests in order to comply with the kit protocol.

At least two wells were seeded as replicates, and at least two photographs were taken for all samples and treatments, stained with dyes or not. The assessment of the cytopathic effect was performed visually.

### 4.8. Western Blot

For Western blot, CCL-1 and HGF cell lines were chosen, and they were seeded in either 6-well plates or 55 mm Petri dishes suitable for cell culture. They were treated with an exposure time of 24 h in an incubator (Panasonic MCO-18AC, Panasonic Healthcare Ltd., Oizumi-Machi, Japan). The treatment was with the selected agents RochC and olympiforin B, and a positive control erufosine (30 µM), a substance proven to cause autophagy [59], kindly provided by Prof. Hans-Jörg Eibl [146]. In addition, a higher concentration of an agent (HirDM90) was added to one of the tests for comparison. The samples were trypsinized, collected, washed with PBS (TS1101, HiMedia, Mumbai, India), centrifuged, and frozen at −80 °C if storage was necessary. Then they were lysed on ice with a buffer containing 100 mM Tris–HCl (648313, Sigma-Aldrich, St. Louis, MO, USA) at pH 8.0, 4% sodium dodecyl sulfate (SDS, L5750, Sigma-Aldrich), 20% glycerol (49779, Sigma-Aldrich), 1% Na_3_VO_4_ (450243, Sigma-Aldrich), 200 mM dithiothreitol (DTT, 3483-12-3, Sigma-Aldrich, Burlington, MA, USA) supplemented with protease inhibitor (05892970001, Roche). The cell lysate was heated to boiling for 15 min, then centrifuged at 13,000 rpm for 10 min at 4 °C. Lysates were subjected to denaturing SDS polyacrylamide gel electrophoresis on acrylamide 4–20% gradient Mini-PROTEAN^®^ TGX™ Stain-Free gels (4568094, Bio-Rad Laboratories, Hercules, CA, USA). The total protein content of the gels of one of the two replicates was photodocumented. The separated proteins were transferred to a polyvinylidene difluoride (PVDF) membrane, a part of the Trans-Blot Turbo Transfer Pack (1704156, Bio-Rad Laboratories), using the Trans-Blot^®^ Turbo™ Transfer System (Bio-Rad Laboratories) according to the manufacturer’s instructions. The blots were then fixed with a blocking solution of 1% casein in PBS (1610783, Bio-Rad Laboratories) and incubated for 2.5 h with specific primary antibodies—rabbit antibodies Atg5, Atg7, and LC3A/B from the Autophagy Antibody Sampler kit (4445, Cell signaling Technology, Danvers, MA, USA) and mouse antibody β-actin (8H10D10, Cell signaling Technology). This was followed by washing and 1 h incubation with a secondary horseradish peroxidase (HRP)-conjugated goat anti-rabbit antibody from the aforementioned kit or horse anti-mouse (7076P2, Cell signaling Technology) for the respective primary antibodies and washing again. Visualization was performed by chemiluminescent reaction with a chemiluminescent substrate (170-5061, Bio-Rad Laboratories). The Western blot was performed in two independent duplicates. Control over the uniformity of sample loading was performed by comparing the protein content of the individual samples with the total protein content for one of the replicates and the actin content, a product of a house keeping gene, for the other replicate. After that optimization, which showed the same tendency in the semi-quantitative results, the results with actin were presented in this work because of their better visual appeal. Densitometric calculation and analysis was performed with Quantity One software version 4.6.9 (Bio-Rad Laboratories).

### 4.9. ELISA for Cytochrome P450

The effect on CYP450 was tested using the ELISA method on the HEPG2 liver cell line. As mentioned, it has weak or absent expression of the CYP450 superfamily, e.g., CYP3A4, CYP1A2, etc. [60], and that low basal activity makes it a good model for studies of CYP inducers [61]. In addition, these cells have the advantage of immortalization due to their cancerous nature. The standard curves after the two ELISA tests (Figure S7) were prepared as a standard dose–response curve, also called a four-parameter logistic (4-PL) curve.

#### 4.9.1. Total Cytochrome P450

Human total cytochrome P450 was examined using the Human Cytochrome P450 ELISA Kit (MBS705506, MyBioSource, San Diego, CA, USA) according to the manufacturer’s instructions. HEPG2 cells were seeded at a concentration of 100,000 cells/mL in a 6-well plate, and the overnight culture was treated with selected *Hypericum* agents. After 24 h of exposure, the protocol was performed according to the kit instructions.

The test principle uses the sandwich quantitative enzyme immunoassay technique. A microplate was pre-coated with an antibody specific for cytochrome P450. Standards and samples were pipetted into the wells, and if cytochrome P450 molecules were present, they were bound by the immobilized antibody. After removing any unbound material, a biotin-conjugated antibody specific for cytochrome P450 was added to the wells. After washing, avidin-conjugated horseradish peroxidase was added to the wells. After washing to remove any unbound avidin-enzyme reagent, a substrate solution was added to the wells, and a color proportional to the amount of cytochrome P450 bound in the initial step developed. The color development was stopped, and its intensity was measured on a microplate reader ELx800 (BioTek Instruments, Inc., Winooski, VT, USA).

#### 4.9.2. Cytochrome P450 3A4

The effect on human cytochrome P450 3A4, the major member of the CYP450 superfamily of enzymes, which is predominantly found in the liver and metabolizes approximately half of the drugs metabolized by CYP450 enzymes, was assayed using the CYP3A4 ELISA Kit (DL-CYP3A4-Hu, DLdevelop, Kelowna, BC, Canada). Sample selection and initial preparation were as for the total cytochrome ELISA. The experiment was performed according to the manufacturer’s instructions. This assay is a sandwich enzyme immunoassay based on the binding of CYP3A4 in standards and samples to a biotin-conjugated antibody specific for CYP3A4. The binding of biotin to avidin conjugated to horseradish peroxidase ensures that after the addition of TMB substrate solution, only those microtiter wells of the plate that contain CYP3A4, biotin-conjugated antibody, and enzyme-bound avidin show a color change. The reaction was stopped by adding sulfuric acid solution, and the plate was read at 450 nm on a microplate reader ELx800 (BioTek Instruments, Inc.). The CYP3A4 concentration was determined by comparing the O.D. of the samples with the standard curve.

### 4.10. In Vivo Skin Irritation Test on Rabbits

Dermal toxicological screening of the chosen two non-toxic active substances was performed on animals. This was carried out according to the European standard ISO 10993-10: 2010 (E): Biological evaluation of medical devices, Part 10 Skin irritation and sensitization tests (6. Irritation tests, 6.3 Animal irritation test) (withdrawn; superseded by ISO 10993-10:2021) [147]. The animal study protocol was approved by the opinion No. 148 of 09.04.2019 of the Ethics Committee of the Bulgarian Food Safety Agency of the Ministry of Agriculture, Food, and Forestry. The Permit to work with experimental rabbits No. 232 was valid until 11.04.2024 and issued on the basis of Article 155, paragraph 7, of the Veterinary Medical Activity Act. The albino rabbits were cared for as specified in ISO 10993-2 (withdrawn; superseded by ISO 10993-2:2022) [148] and Ordinance No. 20 (State Gazette of Bulgaria, No. 87, 9 November 2012). Four square test sites were made on the back of the rabbit by clipping the fur. The samples were applied to 2 of them, while the other 2 were used as a positive control (10–20% SDS) and a negative control (the buffer in which the agents were diluted, i.e., PBS). The exposure time was 4 h, and at the end of it the appearance was checked for erythema (redness) and edema. Additional inspections were performed at hours 24, 48, and 72. The first rabbit per both applied agents did not show any signs of skin irritation, so two more animals per agent were tested, as specified by the ISO standard.

### 4.11. Data Processing and Statistics

At least two wells per concentration (duplicate) were used for the BMD, as well as for the ELISA, live-cell imaging, and Western blot. The CFU and skin irritation test were performed in triplicate—final samples seeded in 3 wells per concentration or 3 animals per agent, respectively. The biofilm assay had 3 to 5 replicates, and the MTT assay had 4 replicates (3–5 or 4 wells per concentration, respectively). The replicates for the time kill assay were provided by the three decimal dilutions of each sample at each hour, from which one replicate was seeded.

The total activity [mL/g] in terms of antimicrobial activity was calculated according to Eloff (2000) [6]. The amount extracted from the plant [expressed as mg/g of dried plant material] was divided by the MIC value [mg/mL].

GraphPad Prism software version 9 (GraphPad Software Inc., San Diego, CA, USA) was used to calculate the IC_50_ values from the MTT assays as in [116], as well as the MTC values, and the ELISA standard curves. The MTC values are, according to ISO 10993-5, the maximum concentration at which at least 70% of the cells are viable (nonlin fit, range) [56]. The 95% confidence interval (CI) of the IC_50_ values is derived from the dose–response curves with a regression coefficient (R^2^) in the range of 0.87–0.99.

## 5. Conclusions

With a view to characterizing them as potential therapeutic agents, a screening of antimicrobial phytoconstituents from *Hypericum* plants with known phytochemical content was performed on selected cell lines, proven models for non-tumorigenic cells, representing different body tissues. All the tested agents had cytotoxicity compared to clinically used drugs such as cisplatin. Most of the ingredients selected as least cytotoxic stimulated the clonogenicity of HaCaT, HEK-293, and CCL-1 lines at doses of 1/2 × IC_50_. A consistent and building assessment selected just two agents—the extract RochC and the compound olympiforin B—to be tested further, as they had outstanding antibacterial activity and satisfactory cytotoxic effect on eukaryotic cells. The concentrations chosen for further research were their MIC values against *S. aureus*—0.625 and 1 µg/mL, respectively. Their IC_50_ values were higher for all five cell lines than these MIC values (except for olympiforin B in HEK-293) and their MTC values were higher for these agents, mostly for the CCL-1 and HGF cell lines. Therefore, at antistaphylococcal doses the agents would be relatively safe for the normal human cells.

RochC and Olympiforin B had MIC values against the cariogenic *S. mutans* of 6 and 3 µg/mL, respectively, values lower than their IC_50_ and even their MTC values for the gingival cells, and have the potential to be developed as oral care products. The MRSA biofilm was more sensitive to the agents, with MBIC values lower than their MIC values. The MBIC of RochC was even one-eighth of the MIC value. The MBIC_50_ was much below the corresponding MIC values for both agents and both bacteria. Olympiforin B at a dose of 1/8 to 1/32 MIC inhibited the relative gene expression of both genes. RochC induced *ica*A, while *ica*D was inhibited by a dose of 1/16 MIC and induced by the lower concentration of 1/32 MIC, showing that the phenotypic inhibition of biofilm formation is not always accompanied by genotypic inhibition of all its related genes. Given that almost all ingredients from the panel of agents initially tested in this study turned out to be bacteriostatic, that quality, in addition to targeting some virulence factor, such as biofilm formation, is a contributing factor for AMR mitigation.

The MIC values for the tested QPS candidate-probiotic lactobacilli were higher than those obtained for *S. aureus*, implying that both agents could be used at anti-staphylococcal doses without significantly compromising the beneficial lactobacilli. These safety and selectivity features are important for topical or systemic anti-staphylococcal treatments, but additional in vivo research to establish their effect on the complex system of host–microbiota–pathogens is to be considered.

There was no cytopathic effect in CCL-1 cells from RochC and olympiforin B treatment, even from doses of 2 × MIC for *S. aureus*. High concentrations of 2 × IC_50_ of another *Hypericum* ingredient also did not cause condensed nuclei, blebs, or other signs of apoptosis, implying that the effect was antiproliferative and cytostatic. Given that other PPAPs cause apoptosis in cancer cells, it is worth elucidating their mechanism of action for their cytotoxic effect on normal cells when applied in high enough concentrations. However, the agents significantly induced Atg5 and Atg7 proteins related to autophagosome formation, while LC3A/B was converted to the lower migrating form—an indicator of autophagy—only by a high concentration of another *Hypericum* constituent and the positive control erufosine.

CYP450 was not induced in HEPG2 liver cells by the agents, with the exception of 3A4 induced by RochC at a dose of 2 × MIC. The selected agents did not irritate rabbit skin in vivo at a dose of even ten times MIC.

The complex evaluation of different aspects of biological activity of the agents shows that RochC and olympiforin B are highly antimicrobial but with a satisfactory gentle effect on the host cells. Therefore, they have potential for further pharmacological development to treat infections caused by certain bacterial strains that are sensitive to them, or as oral care products, or functional foods. However, more research is needed to demonstrate their full therapeutic potential.

## Figures and Tables

**Table 1:** Minimal bactericidal concentrations (MBC) of pure compounds from *Hypericum* species against four pathogenic bacteria (µg/mL and or µM).

Compound	*Staphylococcus aureus*	Methicillin-Resistant *S. aureus* (MRSA)	*Enterococcus faecalis*	*Streptococcus pyogenes*
Olympiforin A	25 (50 µM)	85 (171 µM)	125 (252 µM)	500 (1008 µM)
Olympiforin B	19 (34 µM)	68 (121 µM)	55 (98 µM)	60 (107 µM)
Hyperpolyphyllirin/hyperibine J	94 (194 µM)	- ^1^	- ^1^	- ^1^
Gentamicin (referent antibiotic)	1.9	2.5	72	- ^2^
Penicillin (referent antibiotic)	- ^2^	- ^2^	20	0.25

^1^ Not enough quantity, ^2^ Not tested.

**Table 2:** Minimum inhibitory concentrations (MICs) and MBCs of the extract RochD and the compound Olympiforin B against *Pseudomonas aeruginosa* PAO1 (µg/mL).

Agent	MIC	MBC
RochD	3125	6250
Olympiforin A	1356	2713
Meropenem (referent antibiotic)	1.6	6.4

**Table 3:** MBC/MIC ratio of the agents isolated from *Hypericum* species against pathogenic bacterial species.

Agent	*S. aureus*	MRSA	*E. faecalis*	*S. pyogenes*	*Streptococcus mutans*
RochC ^1^	31	8	16	8	8
RochD	16	- ^2^	32	8	- ^4^
HirDM90	25	16	16	64	- ^4^
RochCM	2	16	8	- ^2^	- ^4^
HirDM100	255	- ^2^	- ^2^	- ^2^	- ^4^
Hyperpolyphyllirin/hyperibine J	47	- ^3^	- ^3^	- ^3^	- ^4^
Olympiforin A	32	7	10	40	- ^4^
Olympiforin B	19	68	14	15	2
Gentamicin	8 ^5^	20	9	- ^4^	- ^4^
Penicillin	- ^4^	- ^4^	8	3	66

^1^ A description of the agents, including the phytochemical content of the three most active agents, is given in [4]. ^2^ MIC could not be determined, e.g., because the extract was cloudy and resembled bacterial growth. ^3^ Not enough quantity. ^4^ Not tested. ^5^ MICs are taken from [4].

**Table 4:** Total activity of the tested extracts and fractions [mL/g].

Extract or Fraction	mg/g Extracted	*S. aureus*	MRSA	*E. faecalis*	*S. pyogenes*	*Y. enterocolitica*	*S. mutans*
RochC	30.45	48,720	3107	6214	1562	0.006	5075
RochD	63.63	12,931	>1632	12,931	816	-	-
HirDM90	58.49	37,494	1500	1500	1500	-	-
RochCM	29.13	2972	187	187	-	-	-
HirDM100	12.38	1263	79	159	20	-	-
BarbD	71.35	- ^1^	-	0.014	-	-	-
RumDBe	51.63	-	-	10	21	-	-
RochM	201.60	-	-	-	81	-	-

^1^ Not tested or MIC could not be determined.

**Table 5:** Mean growth-inhibitory concentrations (IC_50_) of selected agents on five non-tumorigenic cell lines.

Agent	Parameter and Cell Line								
	IC_50_ [µg/mL]	95% CI *	R^2^ **	IC_50_ [µg/mL]	95% CI	R^2^	IC_50_ [µg/mL]	95% CI	R^2^
	**HaCaT**			**CCL-1**			**HEPG2**		
RochC	1.86	1.62–2.14	0.97	5.23	4.34–6.32	0.92	18.05	14.25–22.87	0.90
RochD	3.54	2.92–4.30	0.97	6.05	4.89–7.49	0.92	26.04	21.26–31.89	0.93
HirDM90	1.98	1.61–2.43	0.94	3.48	2.90–4.19	0.93	8.03	7.02–9.18	0.96
Hyperpolyphyllirin/hyperibine J	2.29	1.97–2.66	0.96	7.07	6.27–7.97	0.95	14.33	11.09–18.54	0.87
Olympiforin A	1.33	1.11–1.59	0.95	6.13	4.96–7.58	0.91	6.87	5.14–9.18	0.88
Olympiforin B	1.71	1.59–1.95	0.97	5.50	5.31–5.71	0.98	4.50	3.73–5.42	0.93
	**Human embryonic kidney-293 (HEK-293)**			**Human gingival fibroblasts (HGF)**					
RochC	1.39	1.21–1.59	0.95	9.22	8.28–10.26	0.96			
RochD	2.36	2.16–2.58	0.98	47.83	43.21–52.93	0.97			
HirDM90	0.63	0.32–1.25	0.97	20.65	18.08–23.60	0.95			
Hyperpolyphyllirin/hyperibine J	0.79	0.70–0.89	0.96	12.41	10.83–14.22	0.90			
Olympiforin A	0.69	0.62–0.76	0.97	19.44	18.07–20.90	0.98			
Olympiforin B	0.67	0.60–0.75	0.95	17.98	10.93–29.57	0.98			

* 95% confidence interval. ** Regression coefficient.

**Table 6:** IC_50_ of only the tested substances converted to µM.

Compound	Parameter and Cell Line			
	IC_50_ [µM]	95% CI	IC_50_ [µM]	95% CI
	**HaCaT**		**CCL-1**	
Hyperpolyphyllirin/hyperibine J	4.74	4.08–5.51	14.65	12.99–16.51
Olympiforin A	2.68	2.23–3.20	12.34	9.99–15.26
Olympiforin B	3.65	3.39–4.16	13.08	10.58–16.17
	**HEK-293**		**HGF**	
Hyperpolyphyllirin/hyperibine J	1.64	1.45–1.84	25.71	22.44–29.46
Olympiforin A	1.39	1.25–1.53	39.14	36.38–42.08
Olympiforin B	1.43	1.28–1.60	38.36	23.32–63.09
	**HEPG2**			
Hyperpolyphyllirin/hyperibine J	29.69	22.98–38.41		
Olympiforin A	13.83	10.35–18.48		
Olympiforin B	9.60	7.96–11.56		

**Table 7:** Maximum tolerated concentration (MTC) of the tested active ingredients compared with the antibacterial MIC values [µg/mL].

Agent	MTC on a Cell Line					MIC on Bacteria			
	HaCaT	CCL-1	HEK-293	HGF	HEPG2	*S. aureus*	MRSA	*E. faecalis*	*S. pyogenes*
RochC	0.60	2.55	0.75	7.53	7.52	0.625 ^1^	9.8	4.9	19.5
RochD	0.96	2.91	1.47	41.43	10.34	4.9	- ^2^	4.9	78
HirDM90	0.58	1.84	0.26	17.53	4.21	2.5	39	39	39
Hyperpolyphyllirin/hyperibine J	0.75	4.56	0.46	10.47	5.13	2	8	8	31
Olympiforin A	0.47	3.27	0.41	16.78	2.51	0.78	12.5	12.5	12.5
Olympiforin B	0.52	4.79	0.46	16.06	1.54	1	1	4	4

^1^ The MIC values are taken from [4,29]. ^2^ MIC could not be determined.

**Table 8:** Colony-forming unit (CFU) assay of selected *Hypericum* agents on three cell lines after 48 h exposure to the IC_50_ dose of 72 h and then seeding the cells which formed colonies. The untreated control is normalized as 100% colonies.

**Agent**	**RochC**				**Hyperpolyphyllirin/hyperibine J**				**RochD**			
**Concentration**	Co ^1^	1/2 × IC_50_	IC_50_	2 × IC_50_	Co	1/2 × IC_50_	IC_50_	2 × IC_50_	Co	1/2 × IC_50_	IC_50_	2 × IC_50_
**Colonies per well** ^2^ **for HaCaT cells**	57	71	84	26	55	143	133	58	145	104	83	85
**Percentage**	100	124	147	45	100	259	241	105	100	72	57	58
**Agent**	**Olympiforin B**				**Hyperpolyphyllirin/hyperibine J**				**RochD**			
**Concentration**	Co	1/2 × IC_50_	IC_50_	2 × IC_50_	Co	1/2 × IC_50_	IC_50_	2 × IC_50_	Co	1/2 × IC_50_	IC_50_	2 × IC_50_
**Colonies per well for HEK-293 cells**	104	102	99	80	125	110	91	67	103	117	84	79
**Percentage**	100	97	95	77	100	89	73	54	100	113	82	77
**Agent**	**Olympiforin B**				**Hyperpolyphyllirin/hyperibine J**				**RochC**			
**Concentration**	Co	1/2 × IC_50_	IC_50_	2 × IC_50_	Co	1/2 × IC_50_	IC_50_	2 × IC_50_	Co	1/2 × IC_50_	IC_50_	2 × IC_50_
**Colonies per well for CCL-1 cells**	158	167	62	61	146	162	116	113	41	51	60	146
**Percentage**	100	106	40	39	100	111	79	77	100	124	145	352

^1^ Untreated control. ^2^ Mean of three wells.

**Table 9:** MIC and MBC of RochC and olympiforin B against the cariogenic *S. mutans*, compared to the IC_50_ values for the gingival fibroblasts HGF (µg/mL).

Agent	MIC for *S. mutans*	MBC for *S. mutans*	IC_50_ for HGF	MTC for HGF
RochC	6.25	50	9.22	7.53
Olympiforin B	3.13 (5.57 µM)	6.25 (11.12 µM)	17.98	16.06
Penicillin	0.03	2	-	-

**Table 10:** Biofilm inhibition activity of the chosen agents against *S. aureus* and MRSA.

Bacteria	Agent	Minimum Biofilm Inhibitory Concentration (MBIC)		Median Biofilm Inhibitory Concentration (MBIC_50_)	
		[µg/mL]	[µM]	[µg/mL]	[µM]
*S. aureus*	RochC	0.625 (MIC)	-	0.05 (0.05 to 0.06)	
	Olympiforin B	1 (MIC)	2.14	0.07 (0.06 to 0.07)	0.15 (0.13 to 0.15)
MRSA	RochC	1.25 (1/8 × MIC)	-	0.03 (0.02 to 0.05)	
	Olympiforin B ^1^	0.5 (1/2 × MIC)	1.07	0.05 (0.03 to 0.07)	0.12 (0.09 to 0.17)

^1^ Assessed and published previously.

**Table 11:** Relative gene expression of MRSA after exposure to the selected agents.

Concentration	Agent and Gene			
	RochC		Olympiforin B	
	*ica*A	*ica*D	*ica*A	*ica*D
Control	1	1	1	1
1/8 × MIC	- ^1^	- ^1^	0.37	0.41
1/16 × MIC	2.25	0.8	0.53	0.82
1/32 × MIC	1.03	1.2	0.28	0.95

^1^ Not tested.

**Table 12:** Antimicrobial activity (MIC values, µg/mL) of the tested extracts and fractions on lactobacilli strains.

Agent	Lf98 ^1^	Lf53 ^1^	Lpl.AC11S	Lpl 11	Lpl.L6	Lpl 25
RochC	4	>4	2	4	4	4
Olympiforin B	2	2	2	2	4	2

^1^ The Lf98 and Lf53 designations belong to *Limosilactobacillus fermentum*, and the others belong to *Lactiplantibacillus plantarum*.

**Table 13:** Lactobacilli strains used in the present study.

Strain	Origin ^1^	Species	References
Lf98	Human vaginal isolate	*Limosilactobacillus fermentum*	[137]
Lf53	Human vaginal isolate	*Limosilactobacillus fermentum*	
Lpl.AC11S	White brined sheep cheese	*Lactiplantibacillus plantarum*	
Lpl 11	White brined sheep cheese	*Lactiplantibacillus plantarum*	
Lpl.L6	Katak (traditional Bulgarian food from fermented milk)	*Lactiplantibacillus plantarum*	
Lpl 25	White brined cow cheese	*Lactiplantibacillus plantarum*	

^1^ The selected *L. plantarum* strains were isolated from home-made fermented dairy products, according to traditional technology without utilization of industrial starters.

**Table 14:** Primers and their melting temperatures (Tm) used for the gene expression analysis.

Primers	Sequences	Tm	Reference
*ica*A F	ACACTTGCTGGCGCAGTCAA	69.4 °C	[74]
*ica*A R	TCTGGAACCAACATCCAACA	64.1 °C	
*ica*D F	ATGGTCAAGCCCAGACAGAG	64.3 °C	
*ica*D R	AGTATTTTCAATGTTTAAAGCAA	56.4 °C	
*rho* F	GGAAGATACGACGTTCAGAC	58.8	[145]
*rho* R	GAAGCGGGTGGAAGTTTA	60.3	

## Data Availability

Data is contained within the article and Supplementary Material.

## Supplementary Materials

The following supporting information can be downloaded at: https://www.mdpi.com/article/10.3390/ph18101591/s1, Figure S1: CCL-1 cell line treated with hypertonic buffer. Cells are unstained and stained with Hoechst. Images are at 100× and 200× magnification; Figure S2: Increased number of apoptotic blebs are observed on the cell surface only of CCL-1 cells treated with hypertonic buffer, but not on cells treated with *Hypericum* agents. The magnification is 400×; Figure S3: Cytopathic effect after 5 h of exposure to *Hypericum* agents in CCL-1 cells. The MIC values are for S. aureus. The cells are unstained and stained with Hoechst. Images are at 100× and 200× magnification; Figure S4: Cytopathic effect after 24 h of exposure to *Hypericum* agents in CCL-1. The MIC values are for *S. aureus*. The cells are unstained and stained with Hoechst. The images are at 100×, 200×, and 400× magnification; Figure S5: Cytopathic effect after 48 h of exposure to *Hypericum* agents in CCL-1. The MIC values are for *S. aureus*. The cells are unstained and stained with Hoechst. The images are at 100×, 200×, and 400× magnification; Figure S6: Cytopathic effect after 72 h of exposure to *Hypericum* agents in CCL-1. The MIC values are for *S. aureus*. The cells are unstained and stained with Hoechst. The images are at 100×, 200×, and 400× magnification; Figure S7: Standard curves with linear (a) and logarithmic (b) representation of concentration for human total CYP450 and CYP450 3A4 obtained in the present study; Figure S8: Cell lines not very suitable for colony-forming unit assay. (a) HEPG2 cells, which form a plaque-like colony with difficult-to-count cells; (b) HGF cells, which form a network rather than colonies; (c) HGF cells after MTT salt staining; Table S1: One-way ANOVA analysis for total CYP450; Table S2: One-way ANOVA analysis for CYP450 3A4.
