# Supplementary material for: Polyprenylated Acylphloroglucinols from Hypericum rochelii and Hypericum olympicum—Cytotoxic Effects on Non-Tumorigenic Cell Lines and Antibacterial Potential
Source: Pharmaceuticals (Basel). 2025 Oct 21;18(10):1591. doi: 10.3390/ph18101591 (PMC12566971; doi:10.3390/ph18101591)
Supplement: Supplementary file 1 [file pharmaceuticals-18-01591-s001.zip › pharmaceuticals-3887308-supplementary.pdf]

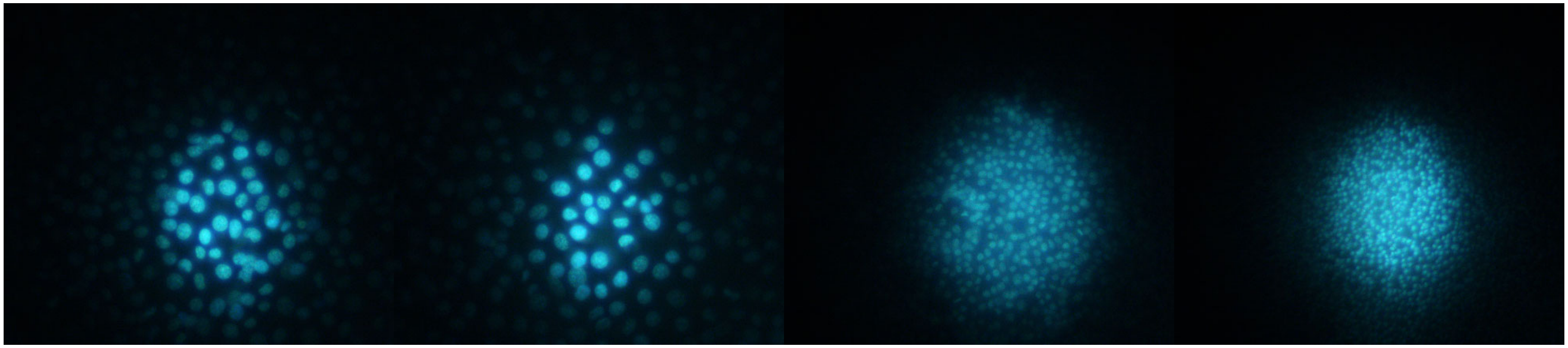

Untreated control, Hoechst

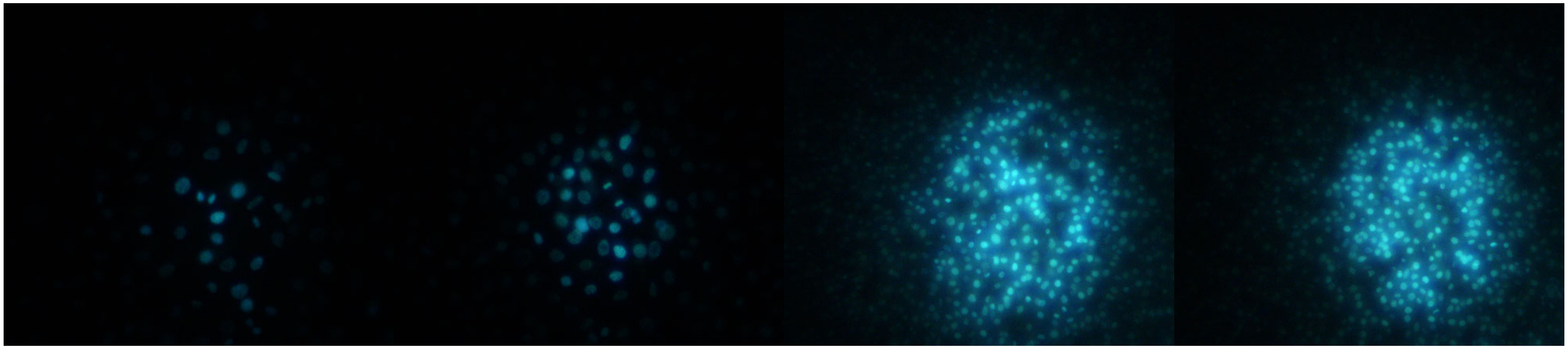

Hypertonic buffer, Hoechst

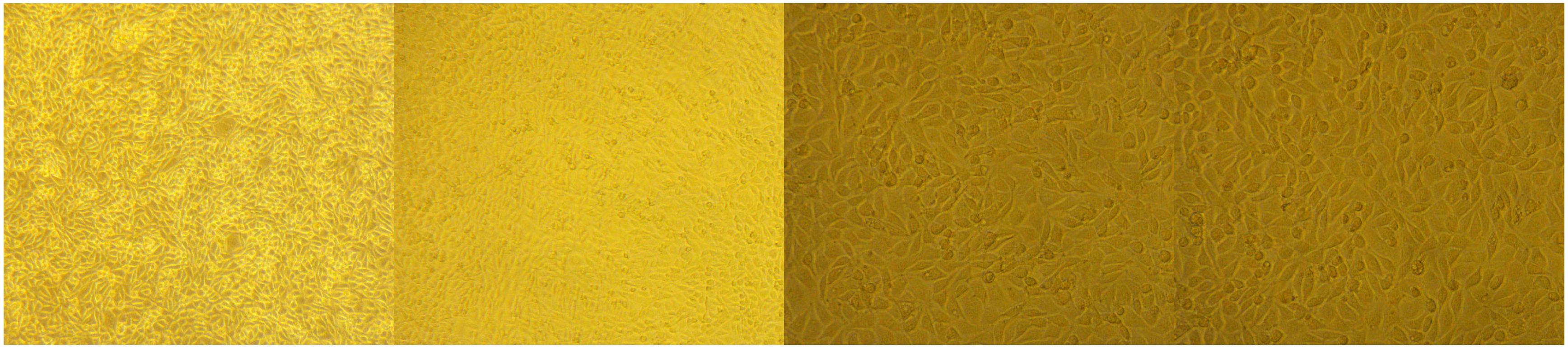

CCL-1, untreated control

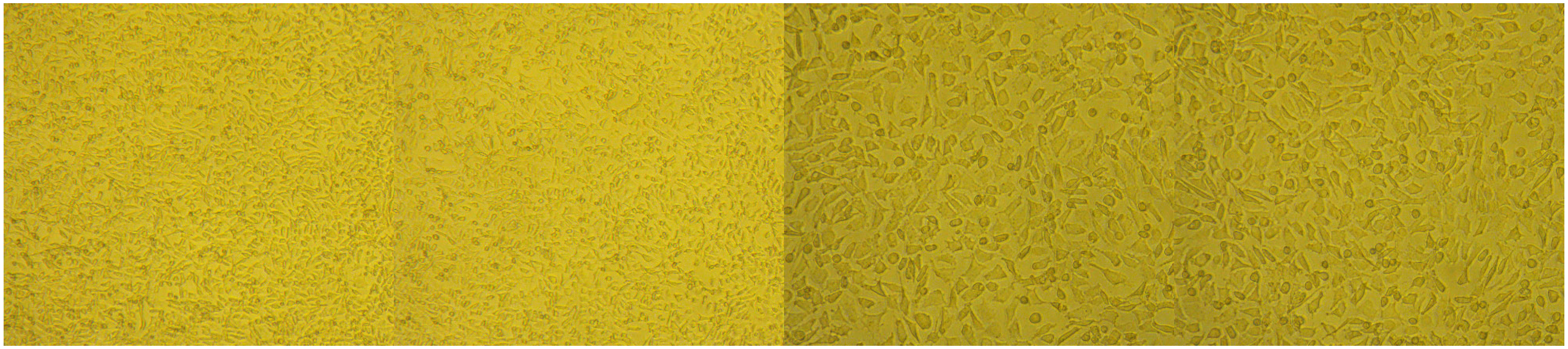

CCL-1, hypertonic buffer

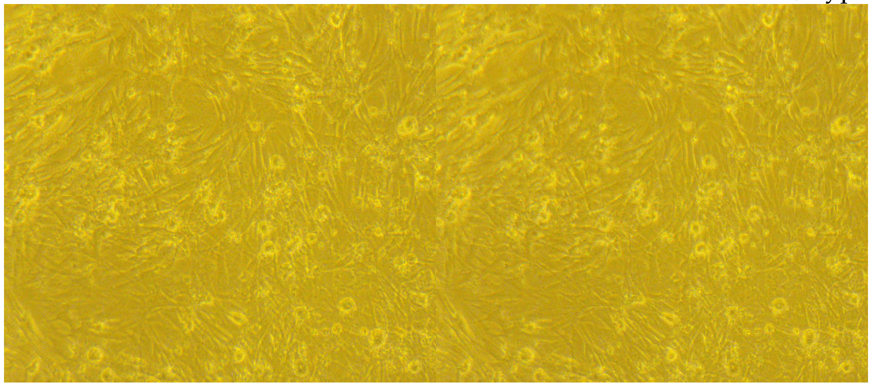

Figure S1. CCL-1 cell line treated with hypertonic buffer. Cells are unstained and stained with Hoechst. Images are at 100× and 200× magnification.

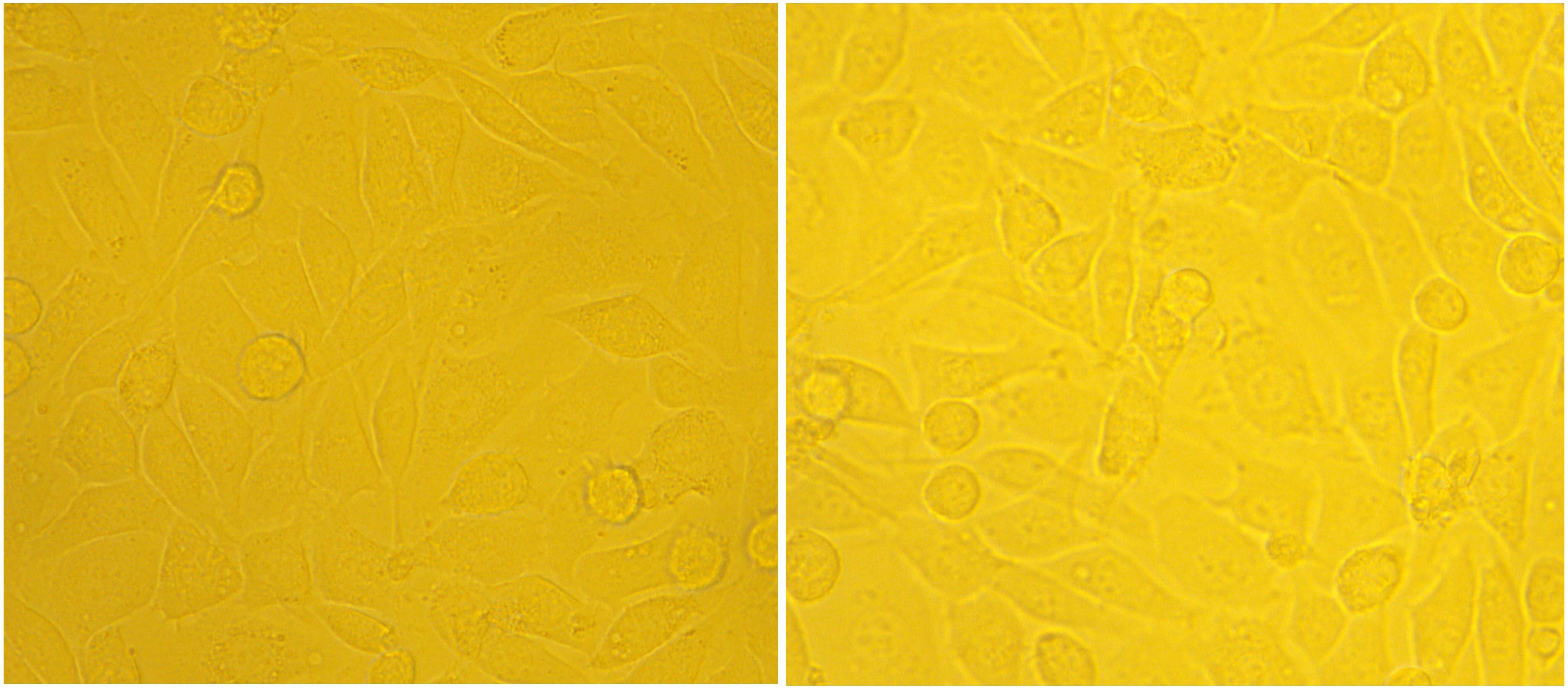

Untreated control

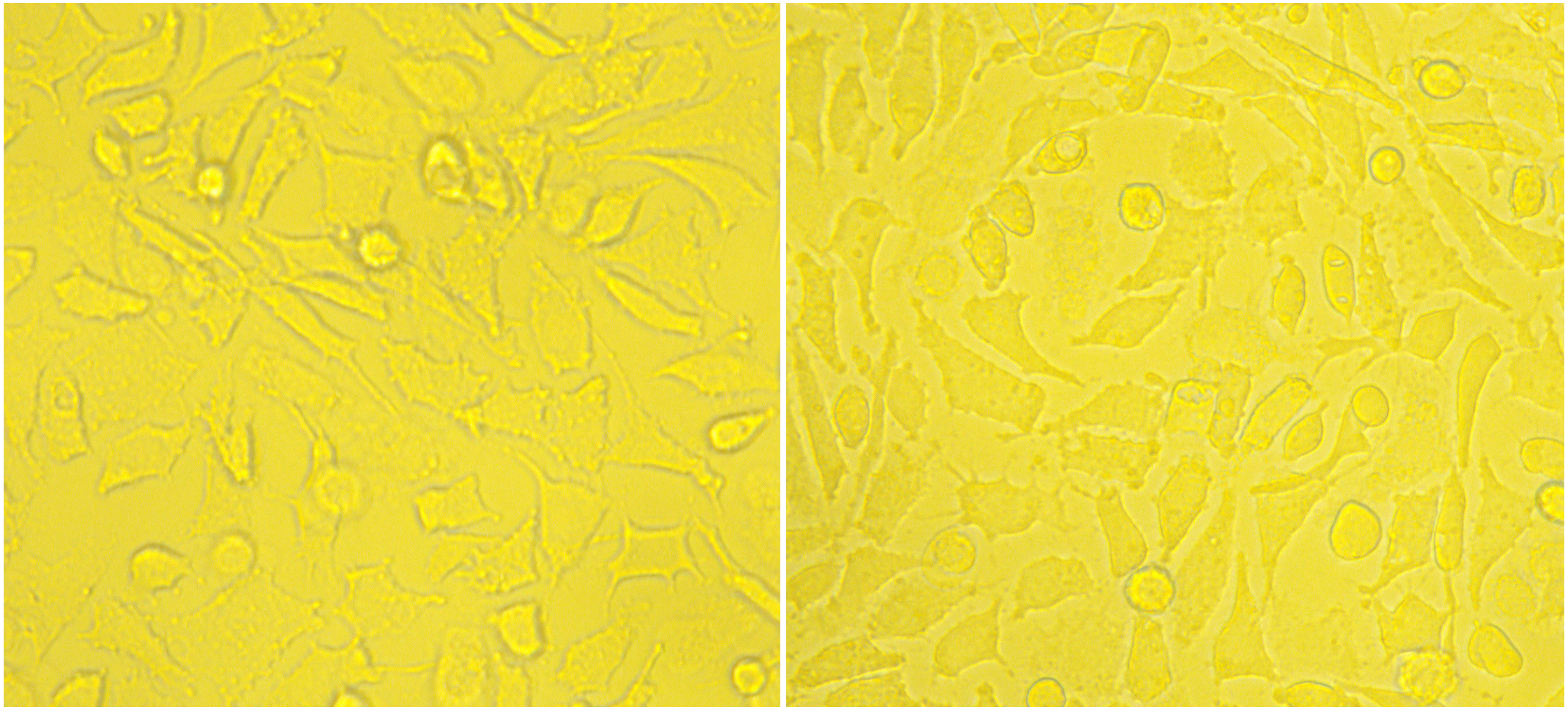

Hypertonic buffer

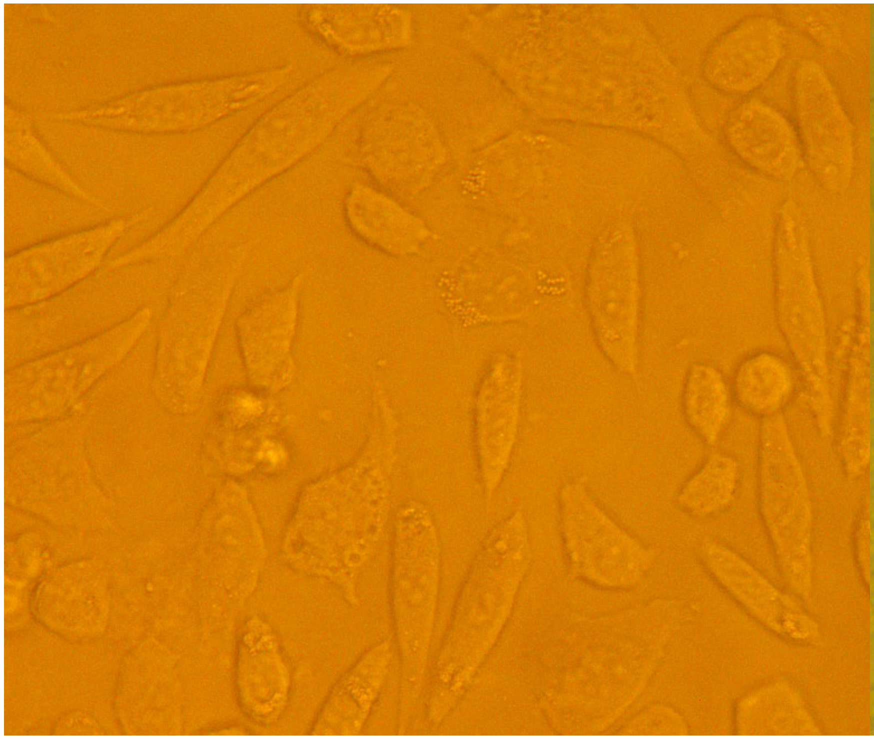

HirDM90 2×IC_{50}, 24 hours

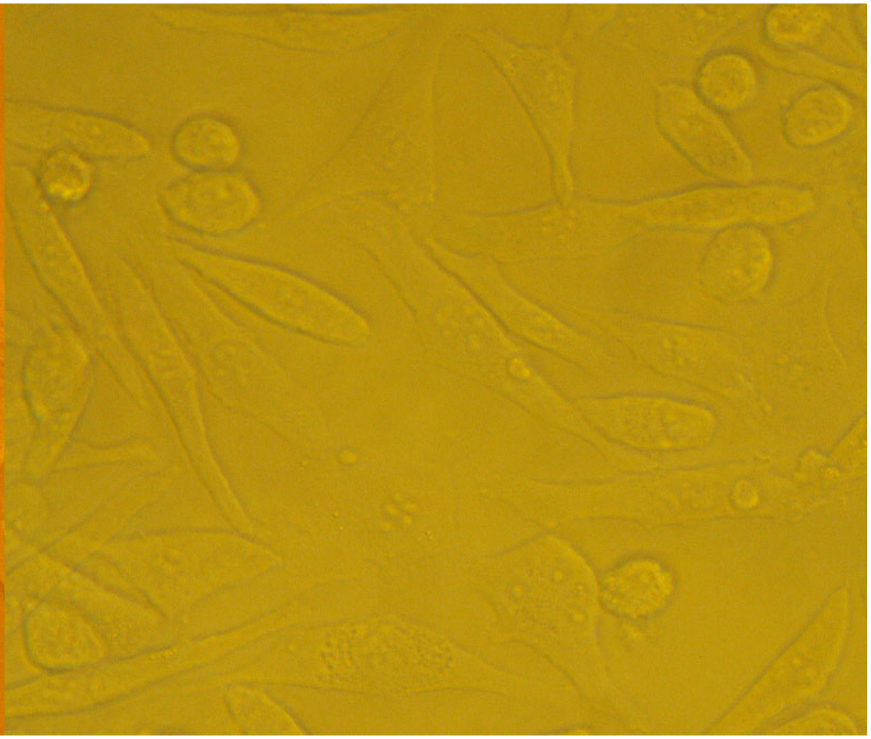

HirDM90 2×IC_{50}, 48 hours

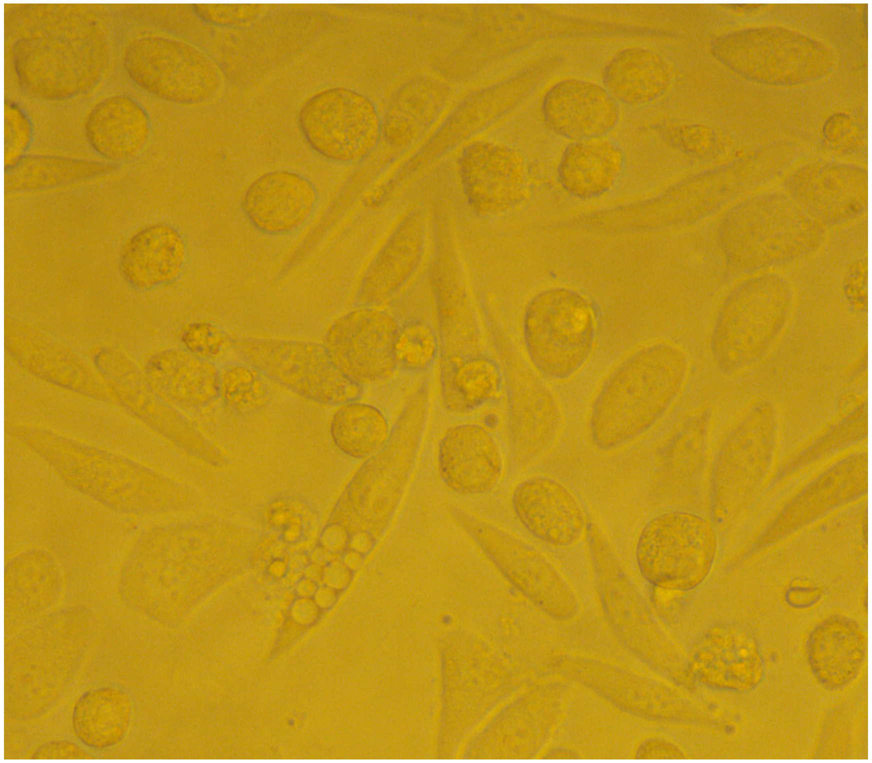

HirDM90 2×IC_{50}, 72 hours

Figure S2. Increased number of apoptotic blebs are observed on the cell surface only of CCL-1 cells treated with hypertonic buffer, but not on cells treated with *Hypericum* agents. The magnification is 400×

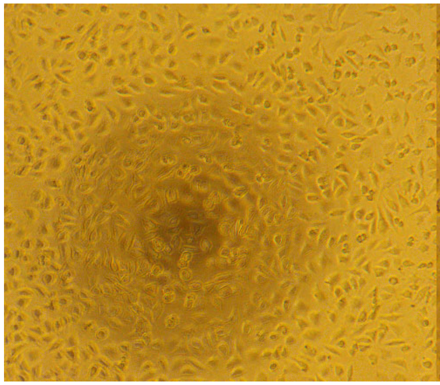

Untreated control

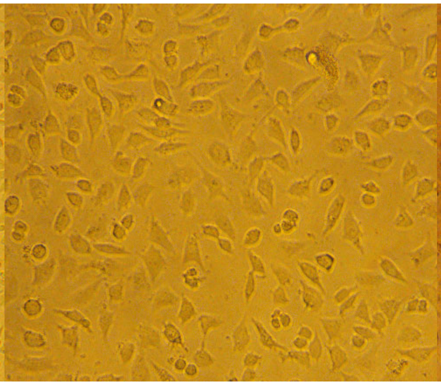

Untreated control

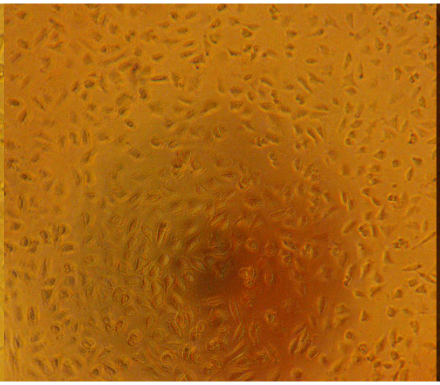

HirDM90 $2IC_{50}$

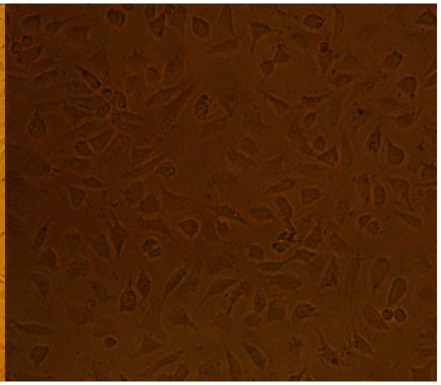

HirDM90 $2IC_{50}$

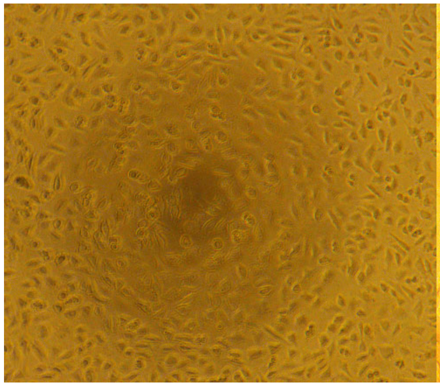

RochC, 2MIC

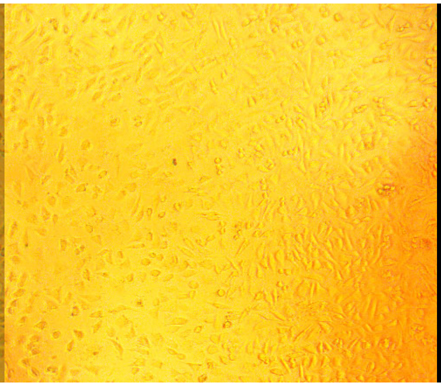

Olympiforin B, 2MIC

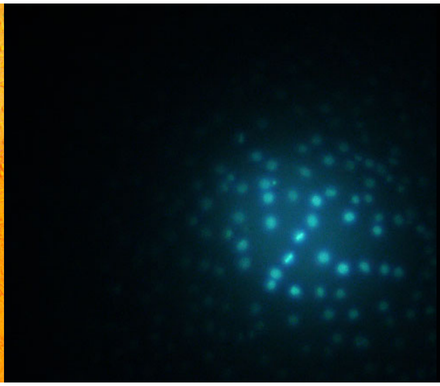

Untreated control, Hoechst

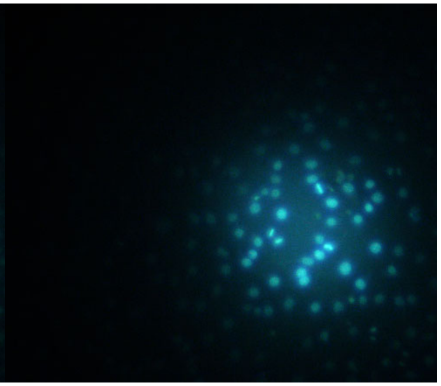

HirDM90 $2IC_{50}$, Hoechst

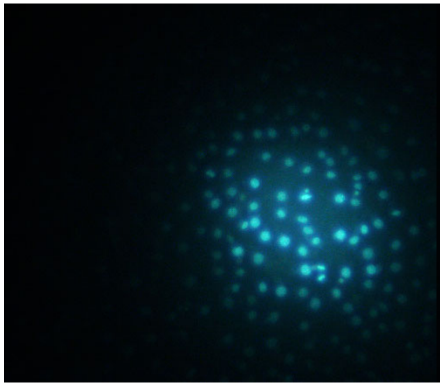

RochC, MIC, Hoechst

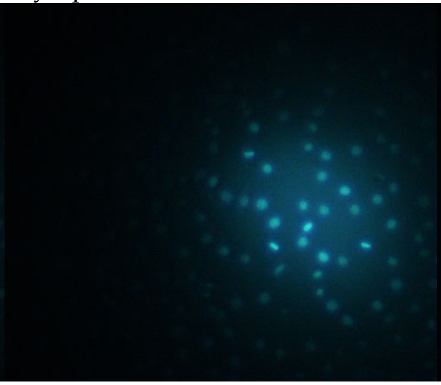

RochC, 2MIC, Hoechst

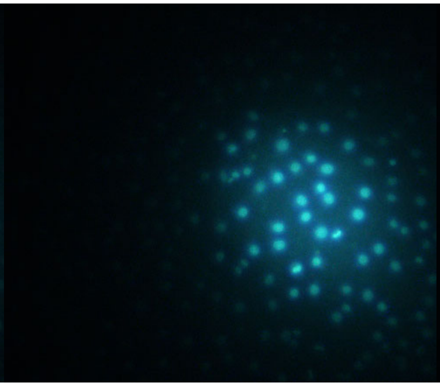

Olympiforin B, MIC, Hoechst

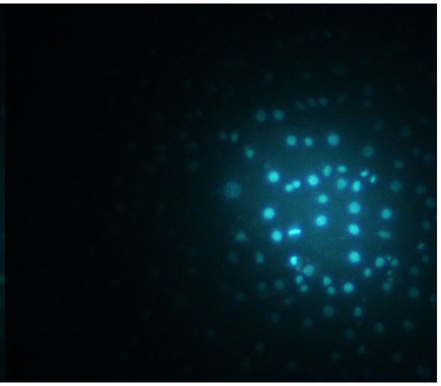

Olympiforin B, 2MIC, Hoechst

Figure S3. Cytopathic effect after 5 hours of exposure to *Hypericum* agents in CCL-1 cells. The MIC values are for *S. aureus*. The cells are unstained and stained with Hoechst. Images are at 100× and 200× magnification.

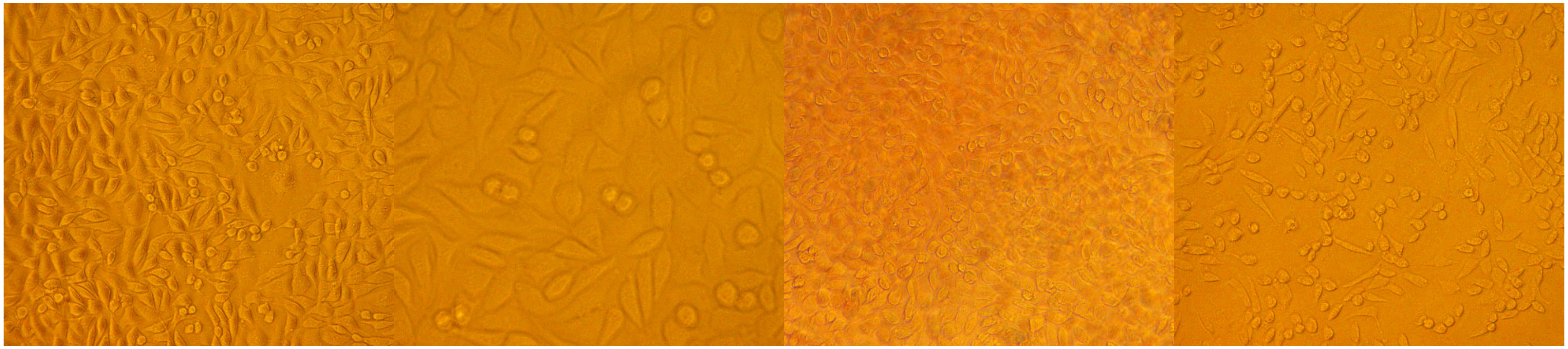

Untreated control | Untreated control | HirDM90 2IC$_{50}$ | HirDM90 2IC$_{50}$

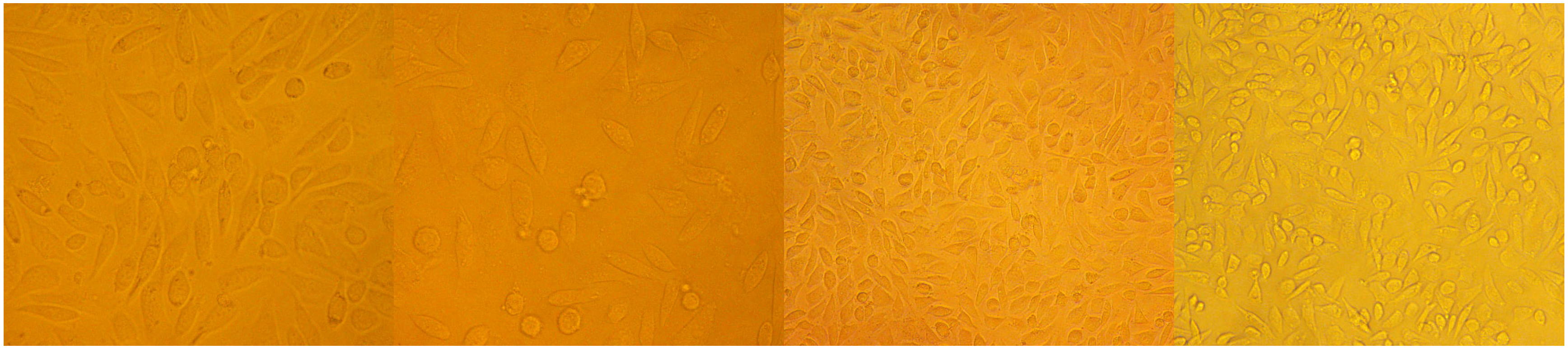

HirDM90 2IC$_{50}$ | HirDM90 2IC$_{50}$ | RochC, MIC | RochC, 2MIC

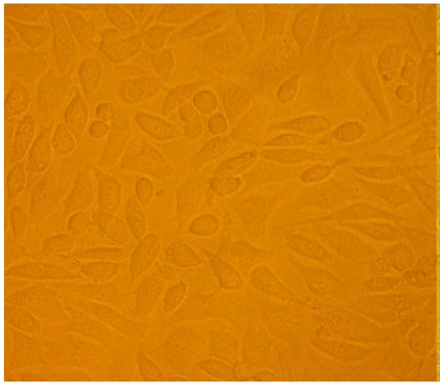
RochC, 2MIC

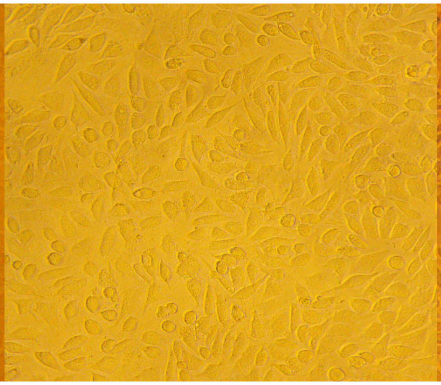
Olympiforin B, MIC

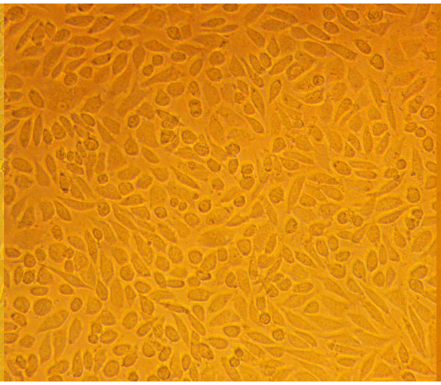
Olympiforin B, 2MIC

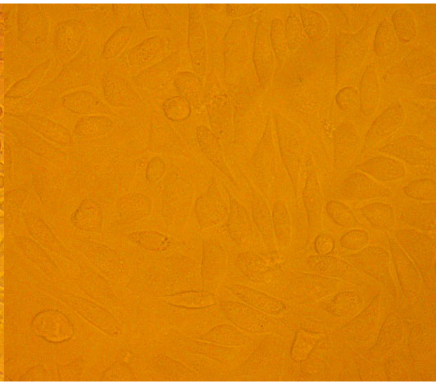
Olympiforin B, 2MIC

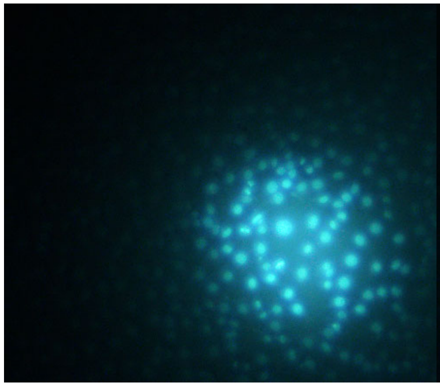
Untreated control, Hoechst

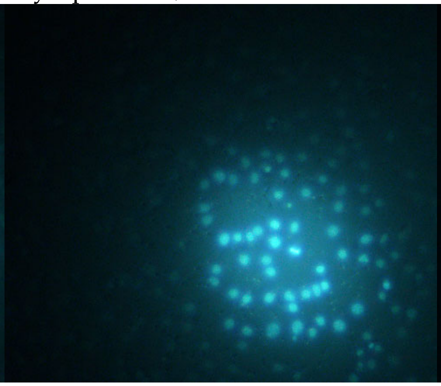
HirDM90 2IC_{50}, Hoechst

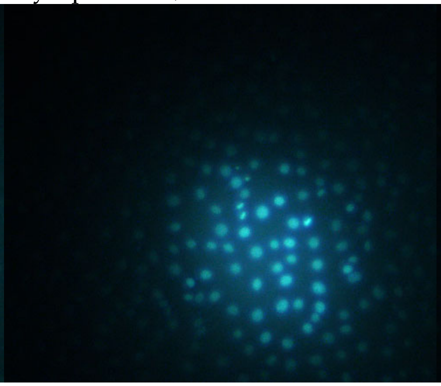
RochC, MIC, Hoechst

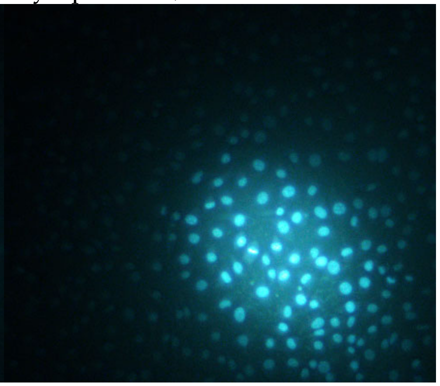
RochC, 2MIC, Hoechst

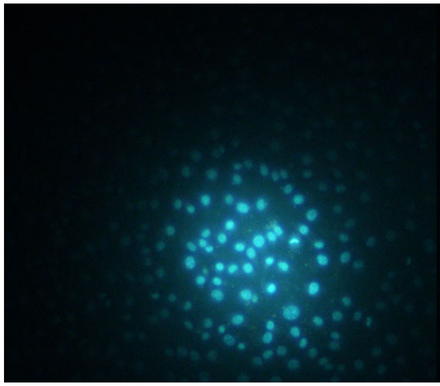
Olympiforin B, MIC, Hoechst

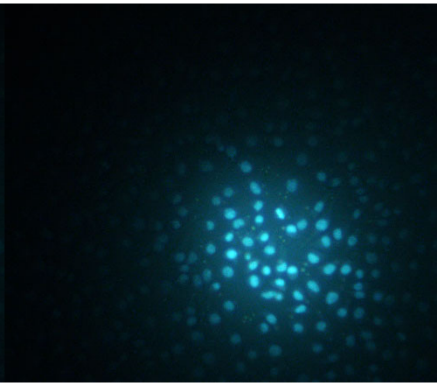
Olympiforin B, 2MIC, Hoechst

Figure S4. Cytopathic effect after 24 hours of exposure to *Hypericum* agents in CCL-1. The MIC values are for *S. aureus*. The cells are unstained and stained with Hoechst. The images are at 100×, 200× and 400× magnification.

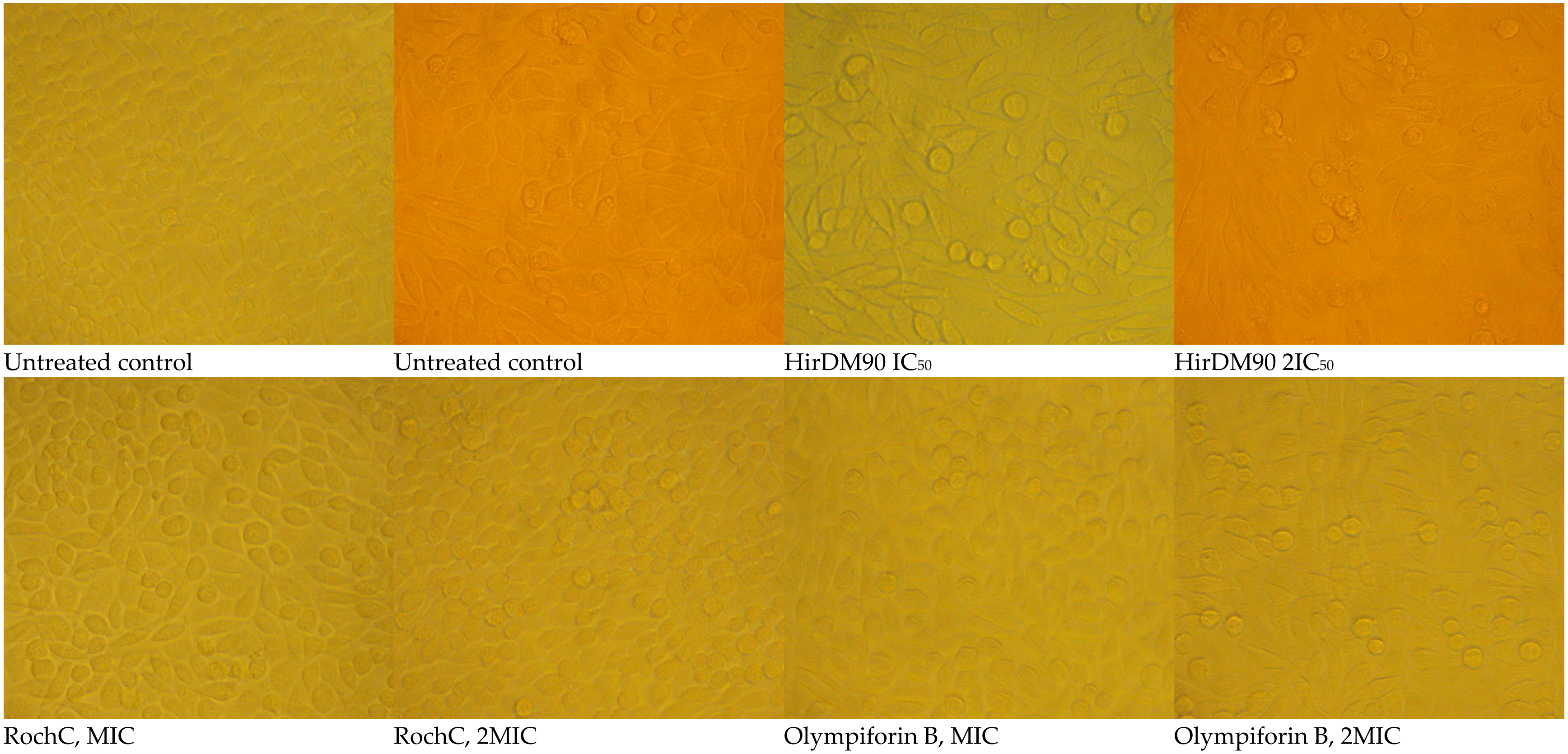

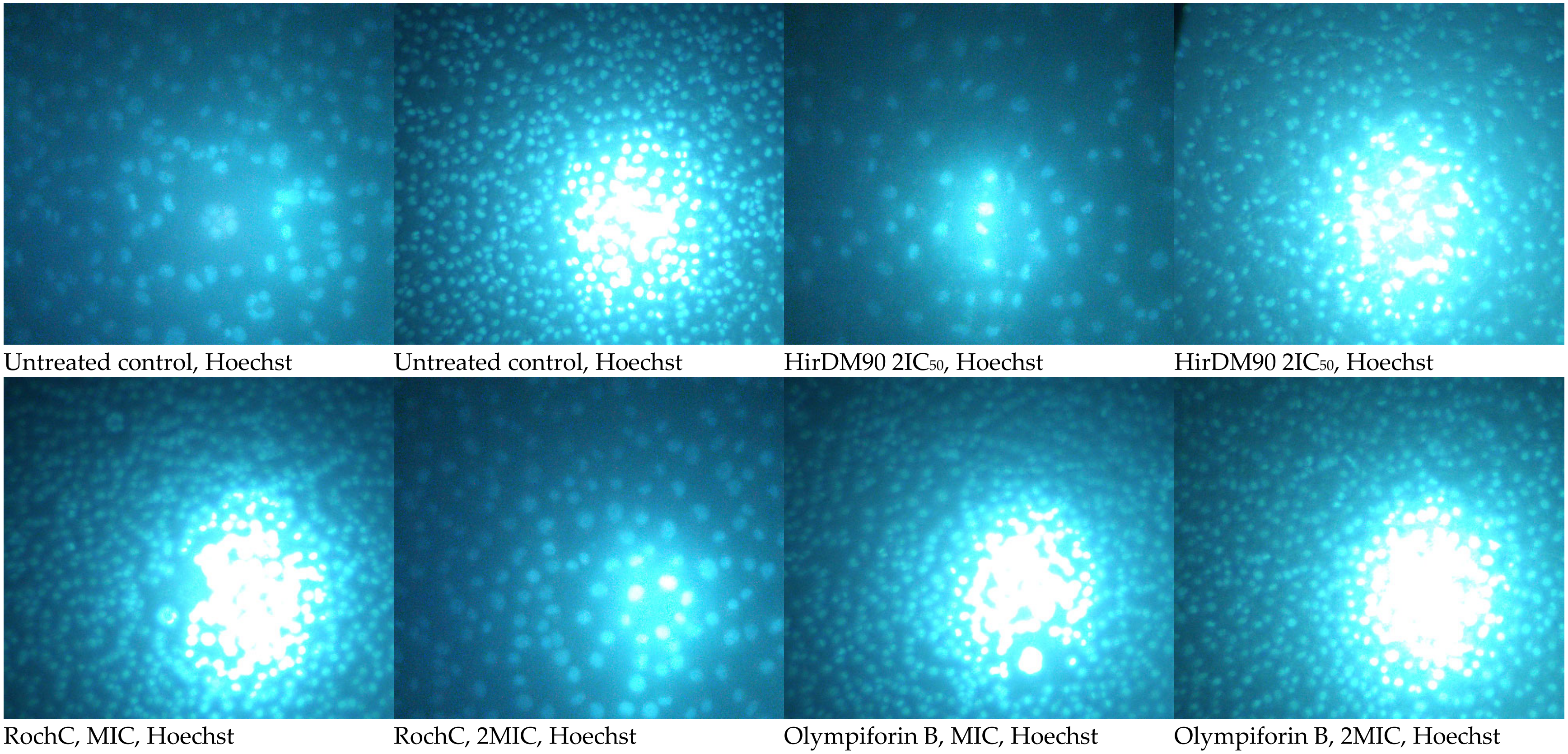

Figure S5. Cytopathic effect after 48 hours of exposure to *Hypericum* agents in CCL-1. The MIC values are for *S. aureus*. The cells are unstained and stained with Hoechst. The images are at 100×, 200× and 400× magnification.

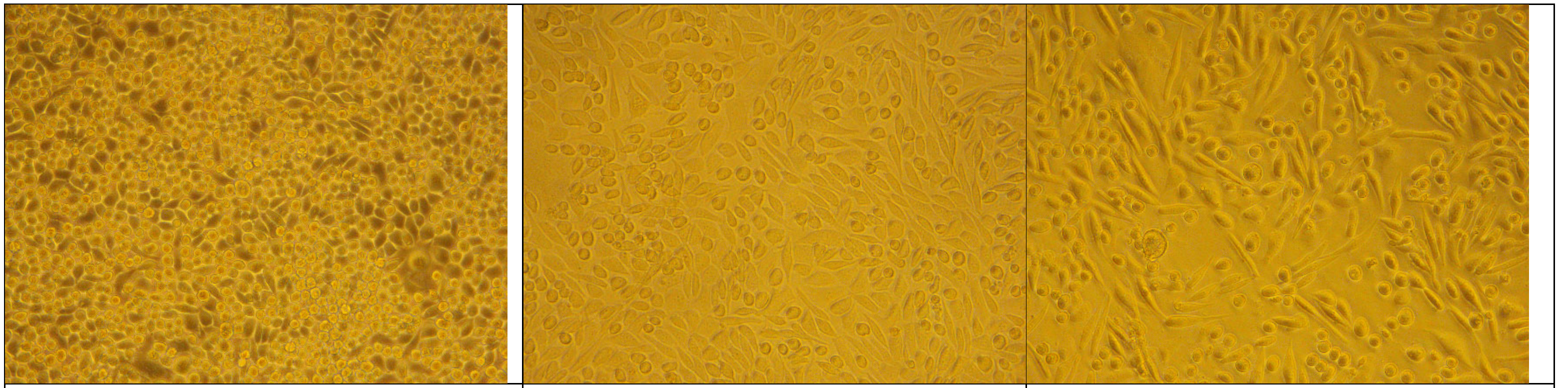

Untreated control | HirDM90 IC_{50} | HirDM90 $2IC_{50}$

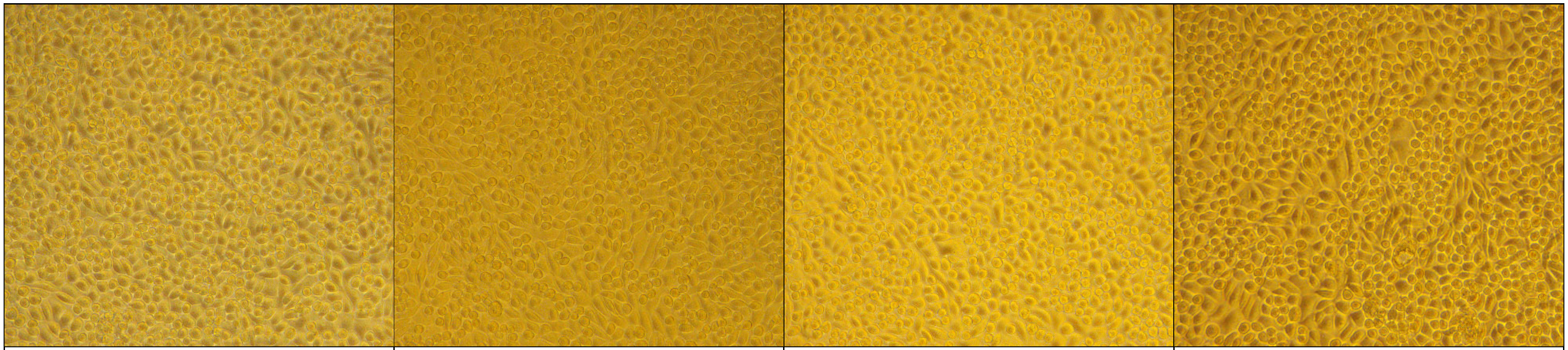

RochC, MIC | **RochC, 2MIC** | **Olympiforin B, MIC** | **Olympiforin B, 2MIC**

Figure S6. Cytopathic effect after 72 hours of exposure to *Hypericum* agents in CCL-1. The MIC values are for *S. aureus*. The cells are unstained and stained with Hoechst. The images are at 100×, 200× and 400× magnification.

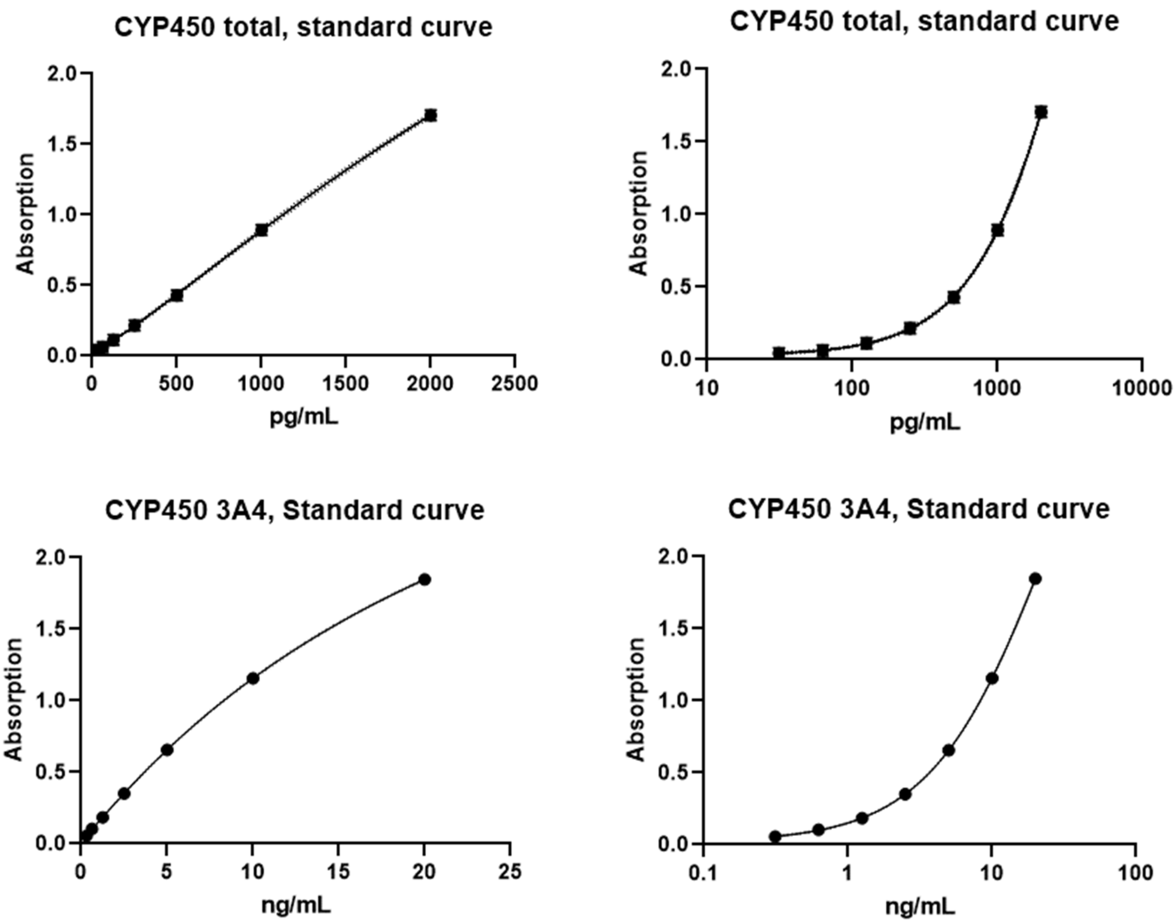

(**a**) (**b**)

Figure S7. Standard curves with linear (**a**) and logarithmic (**b**) representation of concentration for human total CYP450 and CYP450 3A4 obtained in the present study.

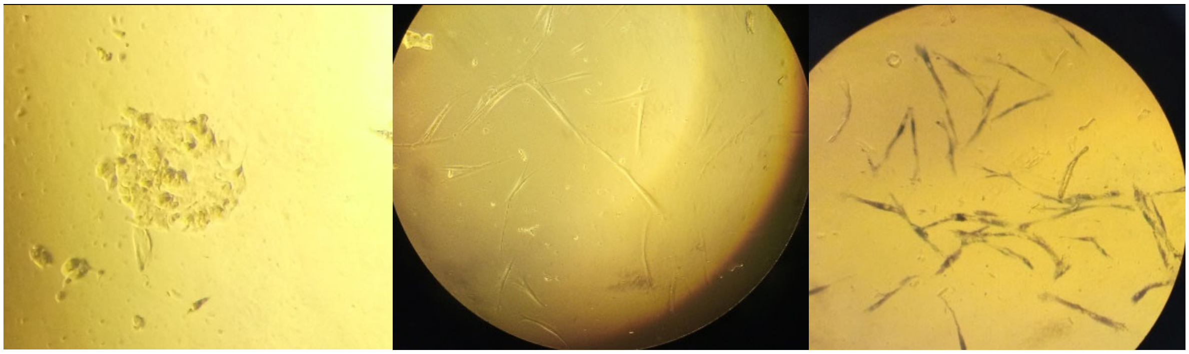

(**a**) (**b**) (**c**)

Figure S8. Cell lines not very suitable for colony-forming unit assay. (**a**) HEPG2 cells, which form a plaque-like colony with difficult-to-count cells; (**b**) HGF cells, which form a network rather than colonies; (**c**) HGF cells after MTT salt staining.

Table S1. One-way ANOVA analysis for total CYP450.

Tukey's multiple comparisons test	Mean Diff.	95.00% CI of diff.	Summary	Adjusted P Value
Co vs. RochC MIC	0.1340	-0.1489 to 0.4169	ns	0.4856
Co vs. RochC 2×MIC	0.1110	-0.1719 to 0.3939	ns	0.6456
Co vs. olympiforin B MIC	-0.01300	-0.2959 to 0.2699	ns	>0.9999
Co vs. olympiforin B 2×MIC	-0.1890	-0.4719 to 0.09385	ns	0.2146
Co vs. HirDM90 2×IC_{50}	-0.3569	-0.6397 to -0.07403	*	0.0176
RochC MIC vs. RochC 2×MIC	-0.02300	-0.3059 to 0.2599	ns	0.9992
RochC MIC vs. olympiforin B MIC	-0.1470	-0.4299 to 0.1359	ns	0.4055
RochC MIC vs. olympiforin B 2×MIC	-0.3230	-0.6059 to -0.04015	*	0.0279
RochC MIC vs. HirDM90 2×IC_{50}	-0.4909	-0.7737 to -0.2080	**	0.0035
RochC 2×MIC vs. olympiforin B MIC	-0.1240	-0.4069 to 0.1589	ns	0.5531
RochC 2×MIC vs. olympiforin B 2×MIC	-0.3000	-0.5829 to -0.01715	*	0.0388
RochC 2×MIC vs. HirDM90 2×IC_{50}	-0.4679	-0.7507 to -0.1850	**	0.0045
Olympiforin B MIC vs. olympiforin B 2×MIC	-0.1760	-0.4589 to 0.1069	ns	0.2628
Olympiforin B MIC vs. HirDM90 2×IC_{50}	-0.3439	-0.6267 to -0.06103	*	0.0209
Olympiforin B 2×MIC vs. HirDM90 2×IC_{50}	-0.1679	-0.4507 to 0.1150	ns	0.2976

L:egend: ns – non significant; * – P ≤ 0.05; ** – P ≤ 0.01.

Table S2. One-way ANOVA analysis for CYP450 3A4.

Tukey's multiple comparisons test	Mean Diff.	95.00% CI of diff.	Summary	Adjusted P Value
Co vs. RochC MIC	0.03973	-0.03211 to 0.1116	ns	0.3535
Co vs. RochC 2×MIC	-0.1389	-0.2108 to -0.06710	**	0.0020
Co vs. olympiforin B MIC	-0.04461	-0.1164 to 0.02722	ns	0.2642
Co vs. olympiforin B 2×MIC	0.0003250	-0.07151 to 0.07216	ns	>0.9999
Co vs. HirDM90 2×IC_{50}	0.1160	0.04416 to 0.1878	**	0.0051
RochC MIC vs. RochC 2×MIC	-0.1787	-0.2505 to -0.1068	***	0.0005
RochC MIC vs. olympiforin B MIC	-0.08434	-0.1562 to -0.01250	*	0.0246

RochC MIC vs. olympiforin B 2×MIC	-0.03940	-0.1112 to 0.03243	ns	0.3602
RochC MIC vs. HirDM90 2×IC_{50}	0.07627	0.004435 to 0.1481	*	0.0387
RochC 2×MIC vs. olympiforin B MIC	0.09433	0.02249 to 0.1662	*	0.0145
RochC 2×MIC vs. olympiforin B 2×MIC	0.1393	0.06743 to 0.2111	**	0.0019
RochC 2×MIC vs. HirDM90 2×IC_{50}	0.2549	0.1831 to 0.3268	****	<0.0001
Olympiforin B MIC vs. olympiforin B 2×MIC	0.04494	-0.02690 to 0.1168	ns	0.2590
Olympiforin B MIC vs. HirDM90 2×IC_{50}	0.1606	0.08877 to 0.2324	***	0.0009
Olympiforin B 2×MIC vs. HirDM90 2×IC_{50}	0.1157	0.04383 to 0.1875	**	0.0052

Legend: ns – non significant; * – P ≤ 0.05; ** – P ≤ 0.01; *** – P ≤ 0.001; **** – P ≤ 0.0001
